# SeHed, a novel gene expression system with stress-evoked hydrogen peroxide elimination property and anti-aging effect

**DOI:** 10.1038/s41392-022-01047-2

**Published:** 2022-07-15

**Authors:** Ning Huang, Honghan Chen, Hui Gong, Haoran Tai, Xiaojuan Han, Xiaobo Wang, Chuhui Gong, Tingting Zhao, Yu Yang, Hengyi Xiao

**Affiliations:** 1 The Lab for Aging Research, National Clinical Research Center for Geriatrics, State Key Laboratory of Biotherapy, West China Hospital, Sichuan University, 1 Keyuan 4 Road, Gaopeng Avenue, Chengdu, 610041 China; 2 Development and Regeneration Key Lab of Sichuan Province, Department of Anatomy and Histology and Embryology, Chengdu Medical College, Chengdu, China; 3 Shaanxi Key Laboratory of Brain Disorders and Institute of Basic and Translational Medicine, Xi’an Medical University, Xi’an, 710049 China; 4 School of Basic Medicine, Dali University, Dali, Yunnan 671000 China

**Keywords:** Senescence, Gene therapy

**Dear Editor**,

The moderate level of reactive oxygen species (ROS) contributes to cellular functions such as proliferation, differentiation, and infection resistance, but the excessive level of ROS causes oxidative damage which underlies the basic mechanism for aging and geriatric diseases.^1^ Therefore, precisely managing cellular ROS levels, meaning to keep redox homeostasis properly, stands as an aim for health and longevity. Actually, this aim is challenging, as it asks not only to restrain excessive ROS accumulation at the right time and right place but also to guarantee proper ROS levels for fitting physiological requirements. Traditional ways for eliminating ROS put more attention on the efficiency and identified a variety of drugs and enzymes with antioxidant properties, known as enzymatic and non-enzymatic ROS scavengers, respectively. However, how to accurately manage the integrative effect of ROS scavengers in vivo remains a problem. Actually, the effectiveness of non-enzymatic antioxidants in vivo is controversial, explained mostly by the uncertainty to reach a given organ with the appropriate concentration or to work there persistently.^2^ As to enzymatic ROS scavengers, although their overexpression in a given place can be achieved, this mode is often unwieldy or harmful to intrinsic physiologic functions in cases.^3^ For this reason, we attempt to develop new ways for in vivo antioxidation, for establishing a high-quality balance between efficiency and safety. As the consequence, we innovated a gene expression system that can limit the excessive ROS accumulation but keep the physiological level of ROS.

This enzymatic scavenger expression system is named as SeHed (Stress-evoked Hydrogen peroxide elimination device), featuring by its stress-evoked response and the moderate capacity for mitochondrial hydrogen peroxide elimination. Popularly speaking, this system works like an automatic fire extinguisher that can put out the burst of ROS under intensive stress conditions. Structurally, SeHed is composed of a stress-sensing promoter portion and a mitochondria-targeted catalase encoding portion (Fig. 1a).

**Fig. 1:**
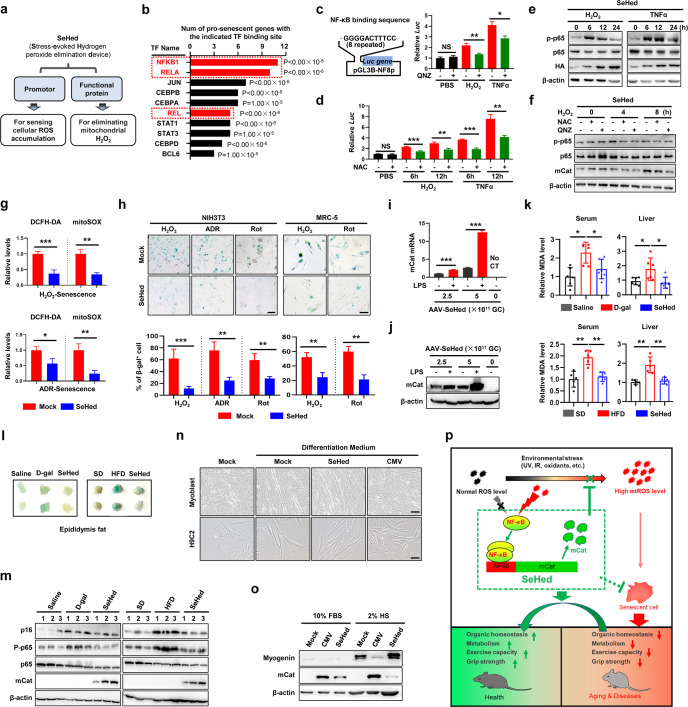
The design, property, senescence alleviation, and age-related phenotype improvement of SeHed. **a** The construction of SeHed. **b** The top ten active transcription factors upregulating pro-senescent genes in oxidative stressed-cellular senescence model predicted by TFactS software. NF-κB transcription factor family, including NF-κB (p52), RELA (p65), and REL (cRel) are remarked as red sign. **c** (left) The construction of luciferase reporter plasmid (pGL3B-NF8p). The NF8p (binding core sequence: NGGGGACTTTCCN), containing eight repeated core sequences, was cloned into the pGL3B plasmid. (right) The dual-luciferase assay was performed in HEK293T cells co-transfected with pRT-L plus pGL3B-NF8p plasmid and treated with H_2_O_2_ (100 μM), TNFα (10 ng/ml), and QNZ (0.5 μM) was applied for 8 h. **d** The dual-luciferase assay was performed in HEK293T cells co-transfected with pRT-L plus pGL3B-NF8p plasmid and treated with H_2_O_2_ (100 μM), TNFα (10 ng/ml), and NAC (2 mM) was applied for 8 h. **e** Representative of western blots for HA (mCat), p65, and p-p65 were shown. NIH3T3 cells were infected with lentivirus for expressing the SeHed (pNF8p-HA-mCAT-puro). Then, cells were treated with H_2_O_2_ (100 μM) or TNFα (10 ng/ml) for different times. **f** Representative of western blots for mCat, p65, and p-p65 in SeHed-expressed NIH3T3 cells treated with H_2_O_2_ (100 μM), NAC (2 mM) or QNZ (0.5 μM) for 4 and 8 h. **g** Cellular ROS and mtROS levels were monitored in H_2_O_2_ or ADR induced-cellular senescence models. The cellular ROS and mtROS levels were labeled by DCFH-DA and mitoSOX, respectively, and quantified by fluorescence microplate reader in Mock- and SeHed-expressed NIH3T3 cells. **h** Representative images of SA-β-gal staining and the ratio of SA-β-gal positive cells were shown. The NIH3T3 and MRC-5 cells were infected with lentivirus expressing the Mock or SeHed, respectively. Then, these cells were incubated with H_2_O_2_, ADR or Rot to induce cellular senescence. **i**, **j** Mice were injected with two doses of AAV9-SeHed virus, respectively (2.5 × 10^11^ GC/mice and 5 × 10^11^ GC/mice). four weeks later, all mice were intraperitoneally injected with LPS (5 mg/kg) for 10 h. (*n* = 3/group). **i** Relative mCat mRNA expression in the liver was analyzed by qRT-PCR assay. **j** Representative images of western blots for mCat in the liver were shown. **k** The MDA level in serum and liver were shown. (left) Mice were divided into Saline, D-gal (100 mg/kg/day) and SeHed (AAV9-SeHed, 5 × 10^11^ GC/mice, plus D-gal (100 mg/kg/day)) groups. (right) Mice were divided into Standard diet (SD), High-fat diet (HFD) and SeHed (AAV9-SeHed (5 × 10^11^ GC/mice) plus HFD) groups (*n* = 5–6/group). **l** Representative images of SA-β-Gal staining of epididymal fat tissue. **m** Representative images of western blots for p16, p65, p-p65, and mCat in the liver. 1, 2, 3 represent mice num. **n** (up) Representative images of myotubes formation in Mock-, CMV-mCAT (CMV)- and SeHed-expressed mouse primary myoblast cells cultured in DMEM medium with 2% HS (Horse Serum) for 3 days. (down) Representative images of myotubes formation in Mock-, CMV- and SeHed-expressed H9C2 cells cultured in DMEM medium with 2% HS (Horse Serum) and 10 μM retinoic acid for 6 days. Scale bars, 50 μm. **o** Representative images of western blots for Myogenin and mCat in Mock-, CMV- and SeHed-expressed H9C2 cells cultured in a medium with 2% HS plus 10 μM retinoic acid for 6 days. **p** Schematic overview of SeHed system in aging and diseases. All data were shown as mean ± SD, *P < 0.05, **P < 0.01, ***P < 0.001; NS no significance

The promoter contains a NF-κB binding element with eight repetitions (NF8p), acting for sensing and responding to external or internal stresses that result in detrimental ROS accumulation. We chose this promoter based on the bioinformatic analysis to an oxidative stress-induced senescence system, in which the NF-κB pathway was the most responsive signal working for pro-senescent gene expression (Fig. 1b, Supplementary Fig. S1). After synthesized according to the conserved sequence of NF-κB binding element,^4^ the performance of this promoter was tested by luciferase reporter assay and GFP reporter assay. As expected, it sensitively responded to H_2_O_2_ and TNFα stimuli (Supplementary Fig. S2a, b), but this response got weakened by NF-κB inhibitor QNZ, p65 siRNA or antioxidant N-Acetyl-L-cysteine (NAC) (Fig. 1c, d, Supplementary Fig. S2c). These results reveal that NF8p is a working promoter for sensing detrimental stresses, especially oxidative distress.

As for the protein-encoding portion of SeHed, we selected the mitochondria-targeted catalase (*mCat*), which was a modified version of human catalase, featuring the deletion of peroxisomal localization signal and the addition of mitochondrial localization signal at the N-terminal (Supplementary Fig. S2d).^5^ We constructed this version of catalase because it is a powerful enzyme for decomposing H_2_O_2_ in cells (Supplementary Fig. S2e), and its distribution in mitochondria facilitates to eliminate excessive H_2_O_2_ immediately after the generation. By linking mCat encoding sequence and NF8p promoter (Supplementary Fig. S2f), the mCat expression of SeHed was quickly induced by H_2_O_2_- and TNFα-stimuli within hours, similar to the response displayed in reporter assays (Fig. 1e, f), and was distributed inside mitochondria (Supplementary Fig. S2i). Strikingly, SeHed effectively lowered cellular ROS levels in stressed cells, both the total and that inside mitochondrion (Fig, 1g, Supplementary Fig. S3d, e). Noticeably, unlike the high and constant pattern of mCat expression driven by CMV promoter, the mCat expression pattern driven by SeHed was relatively low in static condition but elevated quickly in respond to stimuli (Supplementary Figs. S4a, b, S2g, h, S3a, b). Accordingly, our results also shown that, compared with CMV-driven system, the ROS elimination performed by SeHed was moderate, appropriately less than half of the extent by CMV-driven system (Supplementary Fig. S3f–i). These results evidence that SeHed is a stress-sensitized antioxidation system.

Further, we confirmed that SeHed could effectively attenuate oxidative stress-induced cellular senescence. As shown, SeHed lowered the ratio of SA-β-gal positive cells in H_2_O_2_-stressed NIH3T3 cells, decreased the expression of p16, γH2AX, and pro-inflammatory genes, and lengthened cell doubling time (Fig. 1h, Supplementary Fig. S5). Similar results were obtained by using different cells and different senescence models (Fig. 1h, Supplementary Fig. S5). Going ahead, the performance of SeHed in vivo was examined by AAV9-carried SeHed in mice. AAV9-carried SeHed had a wild range of distribution in tissues including liver, heart, skeletal muscle, and fat (Supplementary Fig. S6). At first, the stress-response of SeHed in mice was evaluated by LPS treatment. As shown, both protein and mRNA levels of mCat in the liver increased when LPS loaded (Fig. 1i, j). Subsequently, two kinds of aging-related models were employed for validating the anti-aging effect of SeHed (Supplementary Fig. S7a). One is a progeria model established by chronic D-galactose injection, a way to cause systematic oxidative insults in vivo that also occurs in the natural aging process. As expected, SeHed receded the aging phenotypes, including declined physical activity, malfunctioned liver and kidney, increased SA-β-gal staining, elevated MDA content, and increased the expression of p16 and pro-inflammatory genes (Fig. 1k–m, Supplementary Figs. S7b–d, S8, S10). All these changes imply the tendency toward juvenescence. Another model is high-fat diet (HFD)-induced metabolic disorder, a disease frequently associated with aging. Consistent with the results from progeria model, SeHed not only decreased canonic aging markers, but also improved glucose and lipid metabolism in HFD model (Fig. 1k–m, Supplementary Figs. S8–10). Together, these data support the notion that SeHed is an effective tool for attenuating oxidative stress, alleviating tissue aging and protecting organ function in mice.

One more step forward, we evaluated the influence of SeHed on several physiologic functions in vitro and in vivo. In vitro experiments showed that SeHed did not disturb the differentiation of myoblasts and neural cells, while CMV-driven catalase expression and pharmacological antioxidants (NAC and Mito-tempo) did (Fig. 1n, o, Supplementary Figs. S11a–e, S12a–d). In addition, we performed *Pseudomonas aeruginosa* infection assay, which found SeHed did not impair the innate anti-bacterial function of mice, but NAC at the dose generally used for anti-oxidation damaged this function (Supplementary Figs. S11h, i, S12e–h). The detrimental effect of NAC and Mito-Tempo was also evidenced in vitro, shown as the damaged the bacteria-killing capacity of macrophage (Supplementary Fig. S11f, g). Although more extensive and rigorous investigations need to be conducted, our data preliminarily suggest that compared with traditional antioxidant approaches, either the supplementation of pharmacologic antioxidant or the persistent overexpression of CAT driven by CMV promoter, SeHed has the novel advantage in the aspect of physiologic safety, that makes it having a special potential to develop in vivo study and medical transformation. Moreover, based on its inducible and moderate expression, SeHed may be convenient for digging new clues informative for exploring the more mechanisms on redox homeostasis.

In summary, based on the concept of precise anti-oxidation,^6^ SeHed possesses a special feature for cellular ROS elimination, which means it is adept to eliminate H_2_O_2_ in time, in mitochondria, and in a moderate extent. This makes it facilitating to keep the efficiency and safety of anti-oxidant treatment in a balanced status. SeHed also supports the concept that the relief of the detrimental ROS accumulation and the maintenance of physiological necessary ROS are equally important for antioxidant therapy and for healthy improvement (Fig. 1p). We hope SeHed or its analogs would shed a light on our journey for the goal of precise anti-oxidation, and for medical treatment of multiple human disorders.

## Supplementary information

Supplementary_Materials

Supplementary_Materials

Ethics declarations

Supplementary Figures 1

Supplementary Figures 2

Supplementary Figures 3

Supplementary Figures 4

Supplementary Figures 5

Supplementary Figures 6

Supplementary Figures 7

Supplementary Figures 8

Supplementary Figures 9

Supplementary Figures 10

Supplementary Figures 11

Supplementary Figures 12

## Data Availability

All data that support this study are available from the corresponding author upon reasonable request.

## References

[1] Sies H, Jones DP (2020). Reactive oxygen species (ROS) as pleiotropic physiological signalling agents. Nat. Rev. Mol. Cell Biol..

[2] Forman HJ, Zhang HQ (2021). Targeting oxidative stress in disease: promise and limitations of antioxidant therapy. Nat. Rev. Drug Discov..

[3] West AP (2011). TLR signalling augments macrophage bactericidal activity through mitochondrial ROS. Nature.

[4] Chtarto A (2013). An adeno-associated virus-based intracellular sensor of pathological nuclear factor-κB activation for disease-inducible gene transfer. PLoS ONE.

[5] Schriner SE (2005). Extension of murine life span by overexpression of catalase targeted to mitochondria. Science.

[6] Meng J (2021). Precision redox: the key for antioxidant pharmacology. Antioxid. Redox Signal.

