# Supplementary material for: SeHed, a novel gene expression system with stress-evoked hydrogen peroxide elimination property and anti-aging effect
Source: Signal Transduct Target Ther. 2022 Jul 15;7:235. doi: 10.1038/s41392-022-01047-2 (PMC9283520; doi:10.1038/s41392-022-01047-2)
Supplement: Supplementary file 1 — Supplementary_Materials [file 41392_2022_1047_MOESM1_ESM.pdf]

Supplementary Materials for

SeHed, a novel gene expression system with stress-evoked hydrogen peroxide elimination property and anti-aging effect

Ning Huang, Honghan Chen, Hui Gong, Haoran Tai, Xiaojuan Han, Xiaobo Wang, Chuhui Gong, Tingting Zhao, Yu Yang, Hengyi Xiao.

Correspondence to: hengyix@scu.edu.cn

This PDF file includes:

Materials and Methods
Supplementary Text
Figures. S1 to S12
Table. S1

Materials and Methods

Antibodies and reagents

The following antibodies in our experiments were used: Catalase (ab76024), MHC (Cat No, ab11083), and p16 (Cat No, ab51243) were from abcam; HA (Cat No, 66006-2-Ig) was from proteintech; β-actin (Cat No, bs-10966R) was from Bioss; COX IV (Cat No, 200147) was from ZENBIO; Myogenin (Cat No, A17427) was from Abclonal. p-p65 (Cat No, 3033S) and p65 (Cat No, 8242S) were from Cell Signaling Technology.

The following reagents were used: Rotenone (Sigma-Aldrich, Cat No, R8875), Adriamycin (Selleck, Cat No, S1208), Etoposides (Selleck, Cat No, S1225), Mito-Tempo (Santa Cruz, Cat No, SC-221945), N-Acetyl-L-cysteine (CSN pharm, Cat No, CSN16436), TNFα (novoprotein, Cat No, C008), NGF (novoprotein, Cat No, C060), and Retinoic acid (CSN pharm, Cat No, CSN19418).

The following kits were used: Hieff Canace® Gold High Fidelity DNA Polymerase (Yeasen Biotechnology, Cat No. 10148ES10), ClonExpress MultiS One Step Cloning Kit (Vazyme, Cat No. C113-01), FastPure Plasmid Mini Kit (Vazyme, Cat No. DC201-01), FastPure Gel DNA Extraction Mini Kit (Vazyme, Cat No. DC301-01), Catalase Assay Kit (Beyotime, Cat No, S0051), DCFH-DA probe (Solarbio, Cat No CA1410) and MitoSOX probe (Thermo Fisher, Cat No, M36008), SA-β-Gal staining kit (Solarbio, Cat No G1580,), Oil red O staining kit (Nanjing Jiancheng Bioengineering Institute, Cat No, D027-1-1).

Cell culture

The murine fibroblast (NIH3T3), Human embryonic lung fibroblasts (MRC-5), human embryonic kidney cell (HEK293) and the rat cardiomyocytes (H9C2) were obtained from Shanghai Institutes for Biological Science of Chinese Academy of Science and grown in Dulbecco's modified Eagle's medium (DMEM) supplemented with 10% fetal bovine serum at 37°C in a humidified atmosphere with 5% CO2. Human Primary Fibroblasts (HPF) was obtained from Doc*. Lu* and grown in DMEM supplemented with 20% fetal bovine serum at 37°C in a humidified atmosphere with 5% CO2. The murine macrophage lines Raw264.7 cells were grown in RPMI-1640

supplemented with 10% fetal bovine serum in a humidified atmosphere with 5% CO2. *P*. *aeruginosa* wild-type (WT) strain PAO1 was obtained from Dr. Zhou (Sichuan university, Sichuan, China). The primary myoblasts from about 10-day-old C57BL/6J mice were isolated and cultured following the protocol of Xiaobo wang et al[1]. To induce myogenic differentiation, cells were grown to 60–70% confluence in growth media and then replaced the differentiation medium. Meticulously, primary myoblast cells were cultured in DMEM supplemented with 2% (v/v) horse serum for differentiation, and H9C2 cells were cultured in DMEM supplemented with 2% (v/v) horse serum plus 10 μM retinoic acid for differentiation.

Ethics and Animal procedures

The C57BL/6j mice were purchased from Beijing HFK Bioscience Co., Ltd. All mice were maintained on a 12/12 hours. Eight-week-old male mice were placed for 24 hours into cages and received food and water ad libitum. All animal procedures were performed following the protocol approved by the Institutional Animal Care and Treatment Committee of Sichuan University (Chengdu, China, approval No. 2016064A).

Mice were tail-vein-injected with a dose of 5×10^{11} GC per mouse AAV9-SeHed and AAV9-control in the experiment group and in the control group, respectively. one week later, the mice were injected with D-galactose for the progeria model or fed with high-fat diet (60% fat, 20% protein calories, and 20% carbohydrate calories) for the metabolic disorder model. Meticulously, for D-galactose induced mice progeria model, mice were randomly divided into the Saline (Saline) group, D-galactose (D-gal) group and SeHed group. Saline and D-galactose group mice were injected with AAV9-control virus, and SeHed group mice were injected with AAV9-SeHed virus. One week after AAV injection, the Saline group mice were intraperitoneally injected with saline solution, and D-gal group and SeHed group mice were intraperitoneally injected with D-gal (100 mg/kg/day) for four months. For HFD induced mice metabolic disorder models. All mice were randomly divided into SD group, HFD group and SeHed group. SD and HFD group mice were injected with AAV9-control virus, and SeHed group mice were injected with AAV9-SeHed virus. One week after AAV injection, SD group mice

were fed a standard diet (SD; 10% fat), HFD and SeHed group mice were fed a High-Fat diet (HFD; 60% fat) for four months.

Cellular senescence models

H_2O_2-induced cellular senescence model: a modified H_2O_2 treatment protocol was used. NIH3T3 was trypsinized and suspended in phosphate buffer solution (PBS) at a density of 1×10^6 cells/mL. The cell suspension was transferred to an Eppendorf tube, and then the cells were exposed to 400 μM H_2O_2, incubating at 37℃ for 45 min[2]. Then, the treated cells were then seeded to plate for indicated experiments, and the cells had undergone different treatments as described in individual figure legends. For H_2O_2 induced MRC-5 senescence. MRC-5 were seeded at a 12/24 well culture plates, after cells were cultured for 12 hours, then cells were exposed to PBS contain 400 μM H_2O_2, incubating at 37℃ for 60 min. Then, removing PBS, adding complete medium and continue to culture for 3 days. For drugs (Adriamycin, Etoposides, or Rotenone) induced cellular senescence model[3, 4, 5]. NIH3T3 or MRC-5 were seeded at a 12/24 well culture plates. After culture for 24 hours, the cells were treated with drugs (0.5 μM Adriamycin or 5 μM Etoposides or 0.5 μM Rotenone for 2 days, and continue to culture for 5 days and analyzed later.

Senescence-associated β-galactosidase (SA-β-Gal) staining

For SA-β-Gal staining of cells *in vitro*. Intracellular SA-β-Gal activity was assayed using SA-β-Gal staining kit (Solarbio, Beijing, China, Cat No, G1580) according to the standard protocol, and senescent cells were identified as bluish green stained cells under a phase-contrast microscope. The percentage of β-Gal-positive cells in total cells was determined by counting 1000 cells in 7 random fields, for each group. The results were expressed as the mean ± SD.

For SA-β-Gal staining of tissues (Liver, Lung and Fat) *in vivo*. Tissues were fixed with 10% paraformaldehyde for 24 hours. Then, using gradient concentrations of sucrose for dehydration, and tissues was embedded in OCT, sectioned at 5 μm thickness. After rehydration in PBS, the SA-β-Gal staining was performed using SA-β-Gal staining kit as previous description (Liver incubated 12 hours, Lung incubated 24

hours). In addition, the fat tissue was cut into little pieces, and the SA-β-Gal staining were performed with 12 well culture plate in the staining solution at 37°C for 8-10 hours.

Intracellular ROS, mROS and H_2O_2 level detection

Cellular ROS level was labeled by ROS probe DCFH-DA probe (Applygen, C1300) and detected by fluorescence microscope or flow cytometry. DCFH-DA turns into a green fluorescent molecule called DCF when oxidized by ROS, intracellular ROS levels can be reflected by the fluorescence intensity of DCF[6]. Three independent experiments were conducted.

Cellular mROS level was labeled by mitoSOX probe[7]. To detect mROS levels, cells were loaded with 1 μM MitoSox for 15 min. The MitoSox fluorescence signals was detected by fluorescence microscope. The mean fluorescent intensity of the treated cell population was normalized to fluorescent intensity of untreated cells via image J software (fiji-win64; National Institutes of Health, Bethesda, MD, USA). Three independent experiments were conducted.

Cellular H_2O_2 level were analyzed by protein probe-HyperRed[8]. NIH3T3 was stably expressing HyperRed and SeHed plasmid mediated by lentivirus system. Then, these cells were plated on cover-glass in 2 cm-dishes (Nest, 801001). After cell senescence induced by H_2O_2, the medium was subsequently replaced with 2 ml of phenol-red-free DMEM supplemented with 10% FBS. The cultured dishes were placed in a 37 °C chamber equilibrated with humidified air containing 5% CO2 throughout the microscopy experiment. The fluorescence signals of HyperRed was recorded (excitation at 561 nm, detection at 620 nm) by confocal microscopy. All images were processed using Image J (fiji-win64; National Institutes of Health, Bethesda, MD, USA).

Double luciferase assay and EGFP report assay

For double luciferase assay, 1×10^4 - 3×10^4 HEK293T cells were seeded in 2 ml of growth medium into 24-well plates and incubated under standard cell culture conditions for 24 hours. Then, cells were co-transfected with pTR-L and pGL3B-NF8p as described previously using Neofect™ DNA transfection reagent according to manufacturer's recommendations. After incubation for 24 hours, cells were treated with 100 μM H_2O_2 or

10 ng/ml TNFα, and simultaneously added with antioxidant (NAC) or NF-κB (QNZ) treatment. Following 8-10 hours drug exposure, proteins were isolated and luciferase signals were analyzed using the Dual-Glo Luciferase Assay System (Promega) according to manufacturer's instructions. Others, as to siRNA experiment, cells were transfected by siRNA (sip65, (sense, 5′-GCAUGCGAUUCCGCUAUAATT-3′ and anti-sense, 5′-UUAUAGCGGAAUCG CAUGCTT-3′) was obtained from Sangon Biotech (Shanghai, China)) and co-transfected with pTR-L and pGL3B-NF8p as described previously using Neofect™ DNA transfection reagent according to manufacturer's recommendations. After cells were incubated for 24 hours, cells were treated with 100 μM H_2O_2 or 10 ng/ml TNFα for 8-10 hours. Proteins were isolated and luciferase signals were analyzed using the Dual-Glo Luciferase Assay System (Promega) according to manufacturer's instructions.

For EGFP report assay, $1×10^5$ HEK293T cells were seeded in 1 ml of growth medium into 12-well plates and incubated under standard cell culture conditions for 24 hours. Then, cells were transfected with pNF8p-EGFP as described previously using Neofect™ DNA transfection reagent according to manufacturer's recommendations. After incubation for 24 hours, cells were treated with 100 μM and 200 μM H_2O_2, 0.5 μM ADR or 10 ng/ml TNFα for 24 hours. The EGFP signals were recorded by fluorescence microscope (Olympus, Tokyo, Japan) and analyzed by Image J software (fiji-win64; National Institutes of Health, Bethesda, MD, USA).

Plasmid construction

pGL3B-NF8p plasmid: The NF8p (promotor) fragment containing 8 NF-κB responsive elements is from document[9]. Firstly, the NF8p fragment was obtained by artificial synthesis way (from Tsingke Biotechnology Co., Ltd.). The PCR primers of NF8p fragment: forward primer: 5-TCTTACGCGTGCTAGCCCGGGGCCATGCTCTAGGATCTGAATTG-3, Reverse primer: 5-CAGTACCGGAATGCCAAGCTTGACCGGTGGATCCCGGGC-3. Then, we constructed pGL3B-NF8p plasmid by recombinase replaced the Sma I-Hind III fragment of pGL3B. pNF8p-GFP plasmid: we constructed pNF8p-GFP plasmid by replacing the Cla I-EcoR I fragment of pLVX-IRES-ZsGreen. The PCR primers of NF8p fragment:

forward primer: 5-CCATCGATCGCCATGCTCTAGGATCTGAATTG-3, Reverse primer: 5-CGGAATTCCGACCGGTGGATCCCGGGC-3. The pNF8p-GFP plasmid was obtained by excising the Cla I-EcoR I fragment that containing CMV promotor in pLVX-IRES-ZsGreen and inserting the NF8p fragment into it. pCMV-mCAT: pCMV-mCAT was obtained by inserting the mCat sequence into the MCS sites of pLVX-puro. The mCat fragment was obtained by PCR amplification (pmCAT plasmid, including the mCat fragment, was donated by professor Rabinovitch PS[10]). The primers of mCat fragment: forward primer: 5- TCGAGCTCAAGCTTCGAATTCGCCACCATGCTGTTTAATCTGA-3, Reverse primer: 5- TACCGGTAGAATTATCTAGATCATCCGGACTGCACAAAGG-3. We constructed pCMV-mCAT plasmid by recombinase inserted mCat fragment into the MCS sites (replace the EcoR I-XbaI fragment) of pLVX-puro. SeHed plasmid: SeHed plasmid was obtained by using the NF8p fragment replaced the CMV promotor fragment of pLVX-puro and inserting the mCat sequence into the MCS sites of it. To obtain SeHed plasmid, we firstly constructed the pNF8p-puro by excising the Cla I-EcoR I fragment containing CMV promotor in pLVX-puro and inserting the NF8p fragment into it (primers of the NF8p: forward primer: 5-GCAGAGATCCAGTTTATCGATGCCATGCTCTAGGATCTGAATTG-3, Reverse primer: 5-GTACCGTCGACTGCAGAATTCGACCGGTGGATCCCGGGC-3.). Next, the SeHed plasmid was constructed by inserting the mCat fragment (primers of the mCat fragment: forward primer: 5-CCGGGATCCCGCGACTCTAGAATGCTGTTTAATCTGAGGATCCTGT-3; reverse primer: 5-TACCCGGTAGAATTATCTAGATCATCCGGACTGCACAAAGG -3) into the pNF8p-puro. AAV-SeHed plasmid: AAV-SeHed plasmid was obtained by inserting the NF8p-mCat fragment into the Mlu I site of AAV empty vector (K4 ssAAV.CMV.EGFP.WPREs.SV40PA, from Packgene Biotechnology Co., Ltd). Primers of the NF8p-mCat fragment: forward primer: 5-CATTCGGTACAATTCACGCGTGCCATGCTCTAGGATCTGAATTG-3, Reverse primer: 5-AATTGATTACTATTAACGCGTACACAAAAAACCAACACACAGATCTAATGAA-3.). We constructed the AAV-SeHed plasmid by recombinase for integrating the NF8p-mCat fragment into the Mlu I site of AAV empty vector. The HyperRed plasmid was donated by professor Chen zhouzao (Peking Union Medical College).

Plasmid transfection and lentiviral infection

For the expression of mCat, HEK293T cells were transfected with CMV-mCAT or SeHed respectively, Jet PRIME transfection reagents (PolyPlus, Illkirch, France) was used by according to the manufacturer's instructions. CMV-mCAT and SeHed retrovirus production: HEK293T cells were transduced with CMV-mCAT and SeHed, respectively, and combine packaging plasmids (pSPAX2 and pMDG2) by using transfection reagents. The cell culture supernatant containing virus particles were collected after 72 hours and followed by a 10 min centrifugation at 2000g, and the supernatant was collected and stored at －80℃. The supernatant was used to infect NIH3T3, MRC-5 and HPF cells with10 μg/ml polybrene (Sigma-Aldrich, Carlsbad, CA, USA, Cat No, H9268). The selection of resistant colonies cells was initiated by using 5-20 μg/ml puromycin (Sangon, Shanghai, China, Cat No, 58-58-2) treatment. All experiments involving virus were performed following class II safety procedures.

AAV production and purification

Triple-plasmid transfection using polyethylenimine (PEI, Polyscience) was carried out to produce the recombinant AAV. The transfer plasmids AAV-SeHed; pRep2CapX of AAV-X encoding Rep2 and CapX proteins plasmids; and pHelper were co-transfected into HEK293T cells. The cells were cultured in Dulbecco's modified essential medium (DMEM; Invitrogen, USA) containing 10% fetal bovine serum (Gibco, USA) and 1% S/P antibiotics (Gibco, USA) at 37 °C. When the cells reached 80% confluence, they were transfected in 150 mm plates with 12 μg of pHelper plasmid, 10 μg of AAV pRep2CapX plasmids, and 6 μg of transfer AAV-SeHed plasmids for each plate. At 72 hours post transfection, cells were harvested by 4,000 g centrifugation at 4 °C for 30 min. The pellet was collected and re-suspended in buffer containing 10 mM Tris-HCl, pH 8.0. The suspension was subjected to four freeze-thaw cycles by dry ice/ethanol and a 37 °C water bath. The cell debris was sonicated and then digested with DNase I (200 units in 1.5 ml) for 1 hour at 37 °C. Following centrifugation at 10,000 g for 10 min at 4 °C, the supernatant was collected as the AAV crude lysate. The crude lysate was diluted with 10 mM Tris-HCl pH 8.0 to a final volume of 10 ml and then bottom-loaded to a

discontinuous gradient of 15%, 25%, 40% and 60% Iodixanol in a 39-ml ultracentrifuge tube (QuickSeal, 342414). After ultracentrifugation at 350,000 g and 18°C for 1 hour, 3 ml fractions at lower position of 40% and 0.5ml of 60% upper layer were collected, then repeat the ultracentrifugation at 350,000 g and 18°C for 1 hour one more time and desalted the fractions using a 100 kDa Cutoff Ultrafiltration tube (15 ml; Millipore, USA). The purified AAV were stored at -80 °C before usage. The viral titers were determined by SYBR Green qPCR.

Living imaging

For the EGFP fluorescence imaging *in vivo*. we used the hair shaver for cutting off hair in the back or abdomen of mice. Then, all mice were anesthetized by isoflurane (RWD), and the EGFP fluorescence signals was collected by IVIS Lumina III (PerkinElmer) and the EGFP signals pictures were processed by IVIS Lumina III software.

For the PAO1 luminescence imaging in vivo. Due to the PAO1 has luminescent group. The PAO1 signals in vivo were directly collected by IVIS Lumina III after PAO1 were injected into the mice. The PAO1 luminescence signals pictures were processed by IVIS Lumina III software.

Fasting blood, IPGTT and IPITT

For fasting blood tests, blood glucose levels of all mice were detected via Roche glucometer after 16 hours of fasting. For glucose tolerance test (GTT), mice received one dose of glucose (1 g/kg body weight) via intraperitoneal injection after 16 hours of fasting, and blood glucose levels was measured at 0, 15, 30, 60, 90, and 120 min after glucose injection. For insulin tolerance test (ITT), mice were fasted for 4 hours and then intraperitoneal injected with insulin (0.75 units/kg body weight), and blood glucose levels was measured at 0, 15, 30, 60, 90, and 120 min after insulin injection.

Hematoxylin-Eosin staining Oil red O staining

Liver and Kidney were fixed in formalin, embedded in paraffin and sectioned for H&E staining. Liver sections were fixed in 10% formalin overnight. For the detection of

lipid accumulation, frozen liver sections were stained with oil red O according to a standard protocol (Nanjing Jiancheng Bioengineering Institute, Nanjing, China). Finally, liver sections were imaged at 200×magnification (Olympus, Tokyo, Japan). The area stained with oil red O solution was analyzed with Image J software (National Institutes of Health, Bethesda, MD, USA).

Exercise capacity, grip-strength test and Y-maze test

For mice exercise capacity test. All mice were acclimated to the treadmill system before running test. The exercise regimen commenced at a speed of 15 m/min with an inclination of 5 degrees. Mice were considered to be exhausted and removed from the treadmill following the accumulation of 10 or more shocks (0.5 mA) per minute for two consecutive minutes. The electric shock times were registered within 10 minutes running period.

For grip-strength test. A grip-strength test in mice was performed using a grip-strength meter. Mice were held in front of a horizontal bar, in such a way that only the forelimb paws could grasp the bar. To perform the test, animals were gently pulled back with a steady force until both paws released the bar. Peak tension in grams was recorded for three consecutive trials.

For Y-maze test. The three arms of the Y maze were randomly set as the start arm, the novel arm, and the other arm. Before each test, the novel arm was blocked with a partition, the mouse was put in from the starting arm, and allowed to move freely between starting arm and other arms to adapt for 10 minutes. After 1 hour, opened the new arm, put the mice in platform from the starting arm, let it move freely in the three arms for 5 minutes, the route of mice autonomous movement was recorded. After each test, the Y-maze platform was cleaned with 75% alcohol to remove odors and residues. In the experiment, the starting arm, novel arm and other arms of different mice were randomly arranged. The calculation method is as follows: suppose that the new arm is A, the starting arm is B, the other arm is C, and the movement route of the mice during the test is BACABCBAB. During the test, the mice path passes through 9 arms, and the number of shuttles is 9; consecutive ABC, BCA, and CAB are counted as 1 point. In this example, there are BAC, CAB, ABC, and CBA, that is, the alternate score is 4. The

spontaneous alternation score (%) = [(Number of alternations) / (Total arm entries-2)] *100. In this example, the alternating score rate % = [alternative score 4 / (total number of arms 9-2)] *100%=57.14%.

Biochemical evaluation

Alanine transaminase (ALT) and aspartate transaminase (AST) enzyme activity in serum were determined to evaluate liver injury. Urea and Creatinine levls in serum were determined to evaluate liver injury. Triglyceride and LDL level were evaluated for lipid metabolism. Above blood indexes were detected using a Cobas702 automatic biochemical analyzer (Roche Diagnostics GmbH) in Public Experimental Technology Center, West China Hospital, Sichuan University.

Infection experiments

PAO1 bacteria were grown overnight in lysogeny broth (LB medium) at 37°C with 220 rpm shaking. Next day, the bacteria were collected by centrifugation at 5000×g and resuspended in 20 ml of fresh LB medium, in which they were allowed to grow until the logarithmic phase. Then, the optical density (OD) at 600 nm was measured (1 OD = 1×10^9 cells/ml). Before infection, cells were washed once with PBS, and replaced with antibiotic-free medium. Cells were infected by PAO1 (MOI=10:1; bacteria-cells ratio) for 1 hour. After infection, macrophages were washed 3 times with sterile PBS, extracellular bacteria were removed by addition of 100 µg/ml gentamycin. Phagocytosis was evaluated by counting colony forming unit (CFU)[11]. Bacterial clearance was determined by using the CFU assay after 1 hour infection, following with 5 hours culture in antibiotics medium. For *in vivo* experiments, mice were infected with 2×10^7 CFU of PAO1 (suspended in 200 µl Saline). The PAO1 signals *in vivo* were collected by IVIS Lumina III after infection 3 hours and 8 hours. Mice were sacrificed 8 hours post-infection. The liver and lung were collected for homogenate and CFU assay.

Immunofluorescence

In brief, cells were fixed in 4% (v/v) paraformaldehyde in PBS for 15min at room temperature. Cells were washed thrice with PBS and permeabilized with 0.25% (v/v)

Triton X-100 in PBS for 15 min. After washing thrice times with PBS, cells were blocked in 5% BSA in PBS for 45min at room temperature. Cells were then incubated with primary antibodies in 5% (w/v) BSA in PBS overnight at 4°C. The next day, the cells were washed thrice times with PBS, each for 10 min, followed by incubation with secondary antibody, in 5% (w/v) BSA/PBST for 1 hour at room temperature. The cells were then washed thrice times in PBS, incubated with 1μg/ml DAPI in PBS for 6 min, and washed twice with PBS. The slides were imaged by fluorescent confocal microscope. Cells with more than five visible spots at the expected location were considered positive. The following antibodies were used for immunofluorescence: COX IV (1:400), Catalase (1:300), HA (1:500). Secondary antibodies, anti-mouse Alexa Fluor 594 (Life Technologies, Carlsbad, CA, Cat No, A11005) and anti-rabbit Alexa Fluor 488 (Life Technologies, Carlsbad, CA, Cat No, A11008) were used at 1:1000 dilution. Every experiment was repeated at least 3 times.

Western blotting

Whole cell lysates and tissues lysates were extracted in RIPA lysis buffer (50 mM Tris-base, 1.0 mM EDTA, 150 mM NaCl, 0.1 (w/v) % SDS, 1%(v/v) TritonX-100, 1% (w/v) Sodium deoxycholate, 1 mM PMSF) supplemented with phosphatase inhibitor cocktail (Thermo Scientific). The lysates were rotated at 4°C for 30 min, and supernatants were loaded. The protein concentration was determined by BCA method (CWBIO, Beijing, China, Cat No, CW20011S). The lysates containing 30-50μg of proteins were loaded on the SDS-PAGE gel and separated by electrophoresis, followed by blotting on a PVDF membrane (Millipore, Germany, Cat No, IPVH00010). The target proteins were probed by corresponding primary antibodies with optimized conditions and then incubated with the secondary antibody. Immunological signals were surveyed via electrochemical luminescence method, using Immobilon Western Chemiluminescence HRP substrate kit (Bio-Rad, Hercules, CA, Cat No, 2023) and Fusion Solo Imaging System. Every experiment was repeated at least 3 times, and representative data are shown.

qRT-PCR

Total RNA was isolated from cultured cells using Trizol (Takara, Shiga, Japan) and 1 µg of total RNA were used for reverse transcription by HiScript II Q RT SuperMix for qPCR (+gDNA wiper) (Vazyme Biotech co., ltd, Nanjing, China, Cat No. R223-01), and then quantitative real-time polymerase chain reaction (qRT-PCR) was performed using SYBR Green qPCR Master Mix (Biotool, Shanghai, China, Cat No, B21203). PCR reactions were performed in triplicate and the relative amount of cDNA was calculated by the comparative CT method using the 18s ribosomal RNA sequences as the control. The primer sequences used for qRT-PCR are shown in **supplementary Table 1.** Experiments were repeated three times.

Statistical analysis

Data are presented as means ± SD from at least 3 biological replicates. Statistical analysis was performed using the GraphPad Prism (version 8.0) (GraphPad software, San Diego, CA, USA) using Student t-test. Asterisks indicate statistical significance (*$P<0.05$, **$P<0.01$, ***$P<0.001$).

Reference (methods)

1. Wang, X. B. et al. FTO is required for myogenesis by positively regulating mTOR-PGC-1α pathway-mediated mitochondria biogenesis. *Cell Death Dis*. **8**: e2702 (2017).
2. Han, X. J. et al. AMPK activation protects cells from oxidative stress-induced senescence via autophagic flux restoration and intracellular NAD(+) elevation. *Aging Cell*. **15,** 416-27 (2016).
3. Baar, M. P. et al. Targeted Apoptosis of Senescent Cells Restores Tissue Homeostasis in Response to Chemotoxicity and Aging. *Cell*.**169**, 132-147 (2017).
4. Yang H. et al. cGAS is essential for cellular senescence. *Proc Natl Acad Sci U S A*. **114**, E4612-E4620 (2017).
5. Maurya, N. et al. Low-dose rotenone exposure induces early senescence leading to late apoptotic signaling cascade in human trabecular meshwork (HTM) cell line: An in vitro glaucoma model. *Cell Biol Int*. **40**, 107-120 (2016).
6. Eruslanov. E. et al. Identification of ROS using oxidized DCFDA and flow-cytometry. *Methods Mol Biol*. **594**, 57-72 (2010)

7. Kauffman, M. E. et al. MitoSOX-Based Flow Cytometry for Detecting Mitochondrial ROS. *React Oxyg Species*. **2**, 361-370 (2016).
8. Pei J F, et al. Diurnal oscillations of endogenous H_2O_2 sustained by p66 Shc regulate circadian clocks. *Nat Cell Biol*. **21,** 1553-1564 (2019).
9. Charto, A et al. An adeno-associated virus-based intracellular sensor of pathological nuclear factor-κB activation for disease-inducible gene transfer. *PLoS One*. **8,** e53156 (2013).
10. Schriner, S. E. et al. Extension of murine life span by overexpression of catalase targeted to mitochondria. *Science*. **308**, 1909-1911 (2005).
11. Kannan S, et al. Cholesterol-rich membrane rafts and Lyn are involved in phagocytosis during Pseudomonas aeruginosa infection. *J Immunol*. **180,** 2396–2408 (2008).

Supplementary Text

Figure S1. The NF-κB pathway are activated in both the senescence and acute stress conditions.

a. Representative images of western blots for p-p65 and p65 in H_2O_2- or ADR-induced NIH3T3 and MRC-5 senescence models. **b.** *Il6* and *Tnfα* mRNA levels were analyzed by qRT-PCR in H_2O_2- or ADR-induced NIH3T3 and MRC-5 senescence models. **c.** Representative images of western blots for p-p65 and p65 in H_2O_2-treated NIH3T3 and HEK93T cells for different hours. **d.** *Il6* and *Tnfα* mRNA levels were analyzed by qRT-PCR in H_2O_2-treated NIH3T3 and HEK93T for different hours. **e.** *Il6* and *Tnfα* mRNA levels of liver tissue in Young (2-4 Months) and Old (28-30 Months) mice. **f.** The quantitative data of western bolts for p-p65 in NIH3T3 and HEK93T cells pretreated with NAC (2 mM) or MT (0.5 μM) for 1 hour, and followed by H_2O_2 treatment for different hours. **g**. Representative images of western blots for p-p65 and p65 in NIH3T3 treated with H_2O_2 and incubated in complete medium with NAC (2 mM) or MT (0.5 μM) for 3 days. All data were shown as mean ± SD, * $P < 0.05$, ** $P < 0.01$, *** $P < 0.001$.

Figure S2. The construction of SeHed.

a. The dual luciferase assay was performed in HEK293T cells co-transfected with pRT-L and pGL3B-NF8p plasmid treated with H_2O_2 (100 μM) and TNFα (10 ng/ml) for 8 hours. **b.** Representative images of GFP fluorescence was recorded in transfected-pNF8p-EGFP plasmid HEK293T cells treated with H_2O_2 (100 μM), TNFα (10 ng/ml) and ADR (0.5 μM) for 24 hours. Scale bars, 50 μm. **c.** Representative images of western blots for p65 in HEK293T cells transfected with siRNA (sip65). And the dual luciferase assay was performed in sip65 silenced HEK293T cells co-transfected with pRT-L and pGL3B-NF8p plasmid treated with H_2O_2 (100 μM) and TNFα (10 ng/ml) for 8 hours. **d.** The schematic diagram of catalase catalyzes H_2O_2 to H_2O and O_2. **e.** Mitochondria-targeted catalase was generated by removing the peroxisome localization sequence of wildtype catalase and adding the mitochondrial localization sequence to its' N-terminal. PLS, peroxisome localization sequence; MLS, mitochondrial localization sequence. **f.** The schematic diagram of SeHed plasmid. This plasmid was constructed by replacing CMV with NF8p promotor in pLVX-puro, and mCat sequence was integrated into the MCS site. **g-i.** The Mock, CMV-mCAT and SeHed plasmids were transfected into HEK293T cells, respectively. **g.** Representative images of western blots for mCat. **h.** The Catalase activity of mitochondria. **i.** The Co-localization of mCat and mitochondrial marker protein COX IV. All data were shown as mean ± SD, *** $P < 0.001$.

Figure S3. The expression and the ROS elimination property of SeHed.
a. Representative images of western blots for mCat. Lentivirus system was used to stably express SeHed in NIH3T3, and (up) cells treated with H_2O_2 (100 μM, 200 μM and 400 μM) for 1 hour, and cultured with DMEM medium for 4 hours or 8 hours; (down) cells treated with TNFα (10 ng/ml) for different times. **b.** Representative images of western blots for mCat, p65 and p-p65. The SeHed-expressed NIH3T3 cells treated with H_2O_2 (100 μM), and followed by NAC (2 mM) or QNZ (0.5 μM) treatment for 4 and 8 hours. **c.** The mCat mRNA was analyzed by qRT-PCR. Lentivirus system was used to stably express CMV-mCAT and SeHed in NIH3T3, respectively. Then, cells were pre-treated with H_2O_2 for 4 hours, and followed by NAC treatment for 3 hours and 6 hours. **d.** Representative images and quantitative data of HyperRed fluorescence intensity for cellular H_2O_2 level in H_2O_2-treated Mock-and SeHed-expressed NIH3T3 cells. **e.** Cellular ROS was labeled by DCFH-DA and quantified in Mock- and SeHed-expressed

NIH3T3 cells treated with H_2O_2(treated with 200 μM and 400 μM for 1 hour), Rot (0.5 μM and 1 μM) or ADR (0.5 μM and 1 μM) for 24 hours. **f.** Cellular ROS level was labeled DCFH-DA and monitored by flow cytometry. Lentivirus system was used to stably express Mock, CMV-mCAT and SeHed in NIH3T3, respectively. Then, these cells treated with H_2O_2 (200 μM and 400 μM) for 1 hour, and cultured with fresh DMEM medium for 2, 4, 9, and 24 hours. **g, h.** Lentivirus system was used to stably express Mock, CMV-mCAT and SeHed in NIH3T3 and H9C2, respectively. Intracellular ROS level in untreated cells was labeled DCFH-DA and monitored by flow cytometry. **i.** The Mock, CMV-mCAT, SeHed were transfected into HEK293T, respectively, and co-transfected with HyperRed plasmid. The representative images of HyperRed fluorescence intensity for cellular H_2O_2 level in untreated HEK293T cells. All data were shown as mean ± SD, * $P < 0.05$, ** $P < 0.01$; NS, no significance.

Figure S4. The mCat expression of CMV-mCAT system responses to stress.
a. Representative images of western blots for mCat, p65 and p-p65 in expressed-CMV-mCAT plasmid HEK293T cells treated with H_2O_2 (100 μM) or TNFα (10 ng/ml) for different times. **b.** Representative images of western blots for mCat p65 and p-p65 in expressed-CMV-mCAT plasmid NIH3T3 cells treated with H_2O_2 (100 μM) or TNFα (10 ng/ml) for different times.

Figure S5. SeHed alleviates cellular senescence.
a. Representative images of SA-β-gal staining and the percentage of SA-β-gal-positive cell ratio in Mock- or SeHed-expressed HPF cells. **b.** Representative images of western blots for p16, γH2AX (pS139) and mCat in H_2O_2- or ADR-treated Mock- and SeHed-expressed NIH3T3 cells. **c.** Representative images of western blots for p16, γH2AX (pS139) and mCat in H_2O_2-treated Mock- and SeHed-expressed MRC-5 cells. **d.** The *Il6*, *Il8* and *Ccl3* mRNA levels were analyzed by qRT-PCR in H_2O_2-treated Mock- and SeHed-expressed NIH3T3 cells. **e**. Mock-expressed and SeHe-expressed NIH3T3 were treated with H_2O_2 (100 μM, 200 μM), and cell number was examined for five days. All data were shown as mean ± SD, * $P < 0.05$, ** $P < 0.01$, *** $P < 0.001$.

Figure S6. The distribution and the localization of AAV9-SeHed.

a. The structure information of AAV9-SeHed plasmid. **b.** AAV9-SeHed virus was injected into mice by tail vein injection (5×10^{11} GC per mouse). Four weeks later, mice organs (heart, liver, muscle, intestine (small intestine and colon), epididymis, Fat, brain, spleen, kidney and lung) were separated, and the EGFP signals of tissues above were detected by living imaging. **c.** The co-localization of mCat and COX IV in liver was performed by confocal microscopy.

Figure S7. SeHed improves age-related behavioral phenotypes in progeria mice model.

a. The schematic diagram of animal experiment. **b.** The Electric shock times and exercise distance of Saline, D-gal and SeHed group mice (n=6/group) were detected by treadmill exercise. **c.** The grip strength test of Saline, D-gal and SeHed group mice (n=6/group). **d.** The alternation score of Saline, D-gal and SeHed group mice (n=6/group) were analyzed by Y-maze test. All data were shown as mean ± SD, * $P < 0.05$, ** $P < 0.01$.

Figure S8. SeHed alleviates tissues aging in progeria mice model and metabolic disorder model.

a. Representative images of SA-β-gal staining of liver and lung in Saline, D-gal and SeHed group (n=5-6/group). Scale bars, 50 μm. **b.** *p21* mRNA level of liver and lung was analyzed by qRT-PCR in progeria and metabolic disorder model (n=5-6/group). **c.** Representative images of western blots for p16, p65 and p-p65 in lung. **d.** The senescence associated inflammatory factors mRNA levels of liver and lung were analyzed by qRT-PCR in progeria and metabolic disorder model (n=5-6/group). All data were shown as mean ± SD, * $P < 0.05$, ** $P < 0.01$, *** $P < 0.001$.

Figure S9. SeHed prevents HFD-induced obesity and metabolic dysfunction.

a. Mice weight changes of SD, HFD and SeHed group (n=5-6/group). **b.** Weight of mice after feeding SD or HFD for 16 weeks (n=5-6/group). **c.** Fasting blood glucose levels (n=5-6/group). **d.** Glucose tolerance test and statistics of area under the curve (AUC) (n=5-6/group). **e.** Insulin tolerance test and statistics of area under the curve (AUC) (n=5-6/group). **f.** Mice epididymal fat index (epididymal fat/body; g/g) (n=5-6/group). **g.**

Representative images of HE staining and Oil Red O staining in liver (n=5-6/group). Scale bars, 50 μm. **h, i.** Serum TAG and LDL levels were analyzed. All data were shown as mean ± SD, * *P < 0.05*, ** *P < 0.01*, *** *P < 0.001*.

Figure S10. SeHed alleviates liver and kidney damage in progeria mice model and in metabolic disorder model.

a. AST and ALT activity in serum were analyzed in D-gal-induced progeria model (n=6/group). **b.** Urea and Creatinine levels of serum were analyzed in D-gal-induced progeria model (n=5-6/group). **c.** AST and ALT activity in serum were analyzed in HFD-induced metabolic disorder model (n=5-6/group). **d.** Urea and Creatinine levels of serum were analyzed in HFD-induced metabolic disorder model (n=5-6/group). All data were shown as mean ± SD, * *P < 0.05*, ** *P < 0.01*, *** *P < 0.001*.

Figure S11. Antioxidants impairs capacities for stem cell differentiation and bacterial infection resistance.

a. Representative images of myotubes formation in mouse primary myoblast cells cultured in medium with 2% HS, and followed by NAC (1 mM, 2 mM and 3 mM) and MT (Mito-Tempo, 0.5 μM and 1μM) treatment for 4-5 days. Scale bars, 50 μm. **b.** Representative images of western blots for MHC (myosin heavy chain) in (a). **c.** Representative images of myotubes formation in H9C2 cells cultured in medium with 2% HS plus retinoic acid, and followed by NAC (1 mM and 3 mM) and MT (0.5 μM and 1μM) treatment for 6 days. Scale bars, 50 μm. **d.** Representative images of western blots for myogenin in (c). **e.** Representative images of neurite in PC12 cells cultured in DMEM medium with 2% HS plus Nerve Growth Factor (NGF, 50 ng/ml) for 3 days. Scale bars, 50 μm. **f.** Intracellular PAO1 level was detected by Colony formation assay in Raw264.7 cells pre-treated with NAC or MT for 2 hours and then cells were infected by PAO1 for 1 hour. **g.** Intracellular PAO1 level was detected by colony formation assay in Raw264.7 cells pre-treated with NAC or MT for 2 hours, and then cells were infected by PAO1 for 6 hours. **h.** The PAO1 level *in vivo* was detected by living imaging in control group and NAC group (IP, 200 mg/kg; n=4-5/group) infected PAO1 for 3 hours and 8 hours. **i.** PAO1 level in liver and lung (n=4-5/group) in (h) were detected by colony

formation assay. All data were shown as mean ± SD, * $P < 0.05$, ** $P < 0.01$; NS, no significance.

Figure S12. SeHed does not impairs cell differentiation *in vitro* and bacterial infection resistance *in vivo*.

a. Representative images of western blots for mCat in primary myoblast cells expressing SeHed by lentivirus infection. **b.** Representative images of western blots for MHC in the Mock- and SeHed-expressed primary myoblast cells cultured in medium with 2% HS for 4-5 days. **c.** Representative images of western blots for mCat in H9C2 cells expressing SeHed by lentivirus infection. **d.** Representative images of neurite in Mock-, CMV-mCATes (CMV)- and SeHed-expressed PC12 cells cultured in DMEM medium with 2% HS and Nerve Growth Factor (NGF, 50 ng/ml) for 3 days. Scale bars, 50 μm. **e.** Representative images of abdomen EGFP signals was detected by living imaging. Mice were divided into two groups: control group (n=4) and SeHed group (n=4), SeHed group mice were injected with AAV9-SeHed (5×10^{11} GC) by tail vein injection for four weeks. **f.** Representative images of PAO1 level *in vivo* of PAO1 infected mice by intraperitoneal injection after 3 hours and 8 hours. **g.** The PAO1 level in liver and lung were analyzed by clone formation assay. NS, no statistical significance. **h.** Representative images of western blots for mCat in liver in (e).

Table. S1. The primers for the experiment.

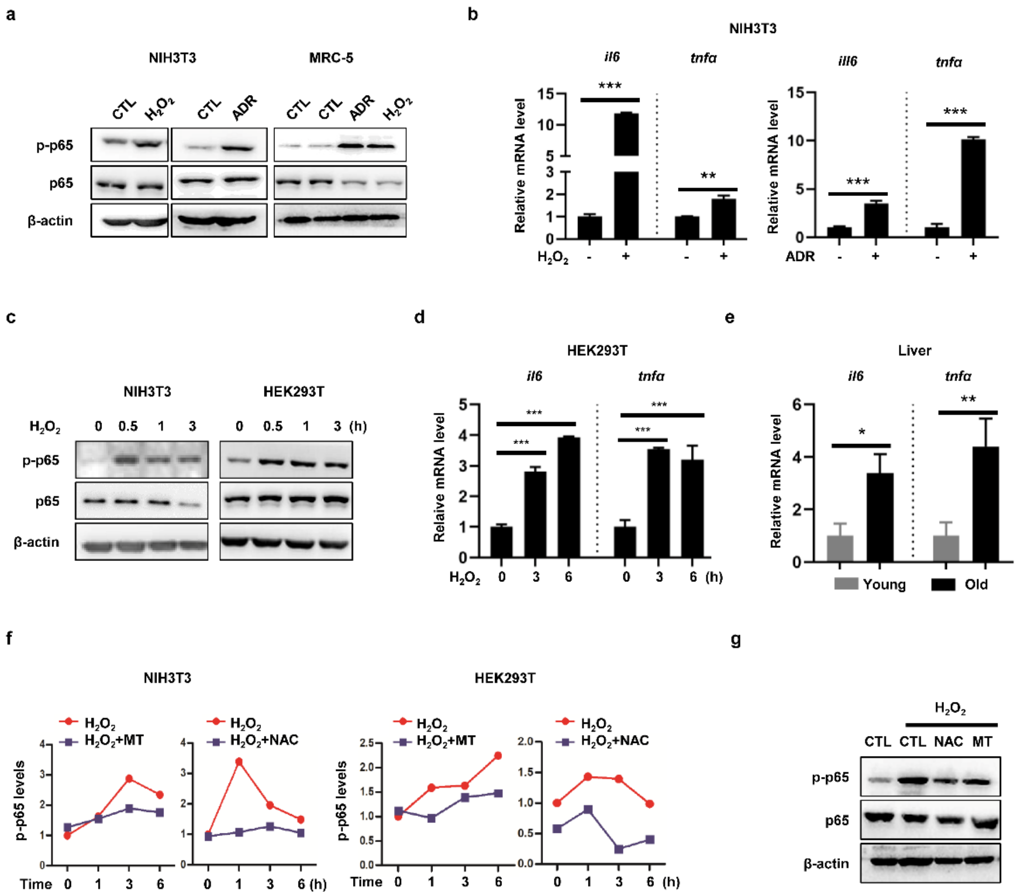

Figure. S1. The NF-κB pathway are activated in both the senescence and acute stress conditions.

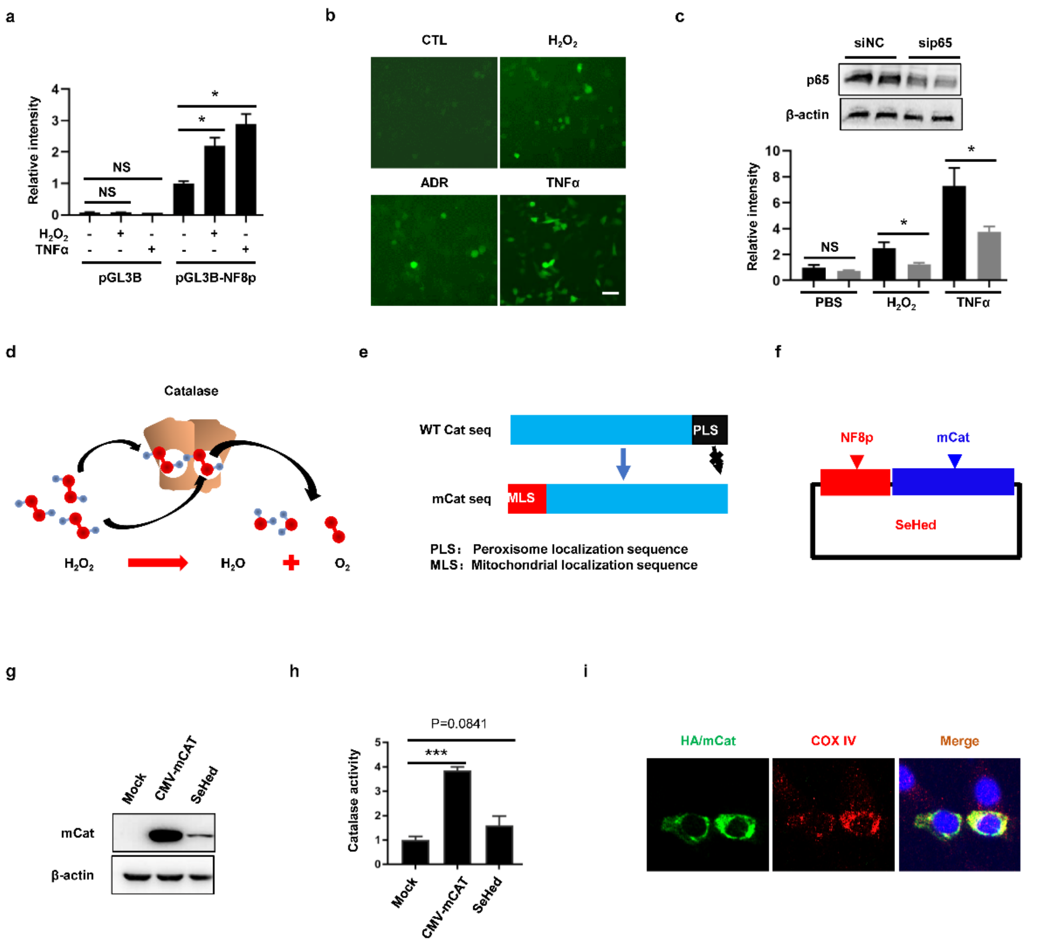

Figure. S2. The construction of SeHed.

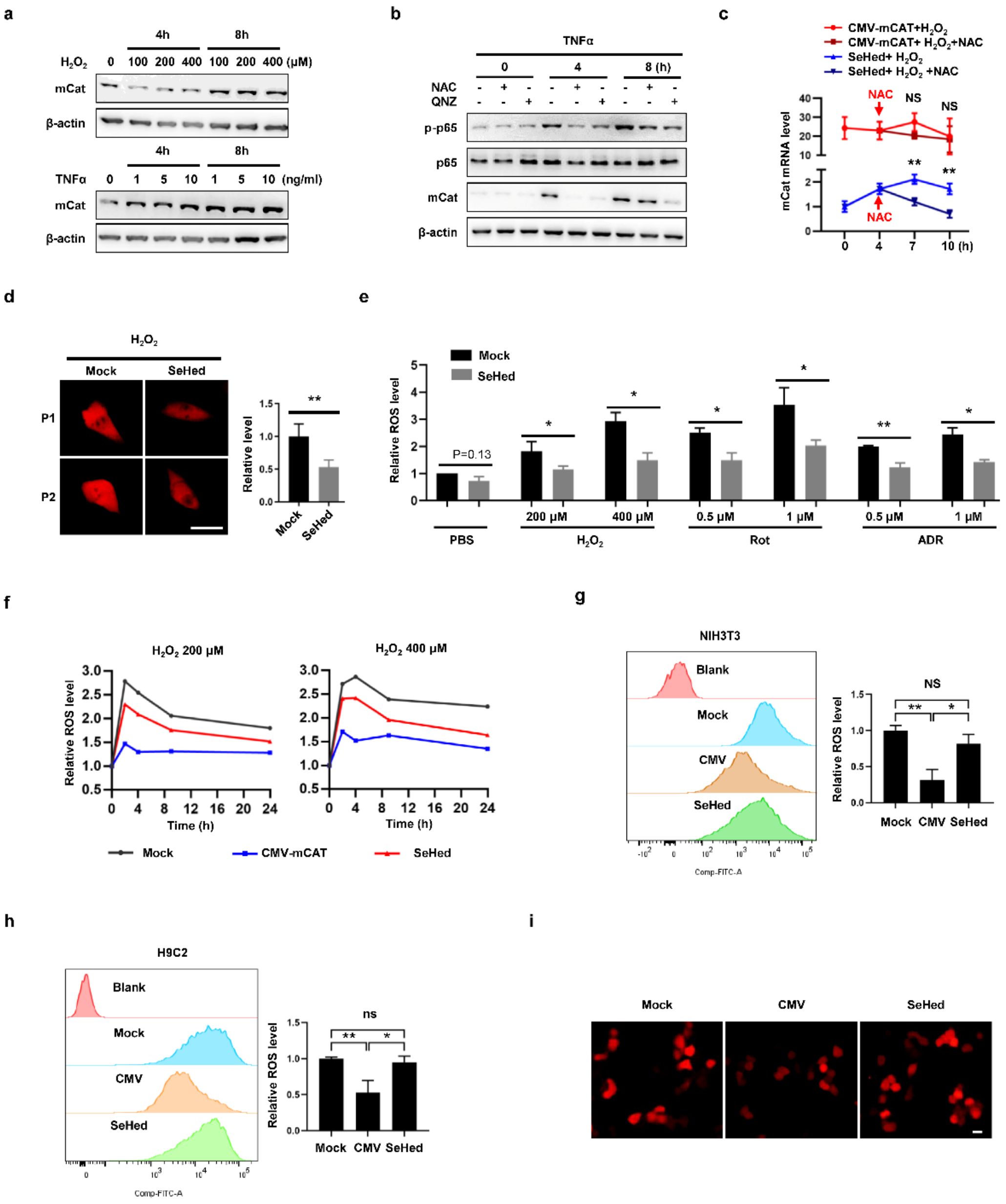

Figure. S3. The expression and the ROS elimination property of SeHed.

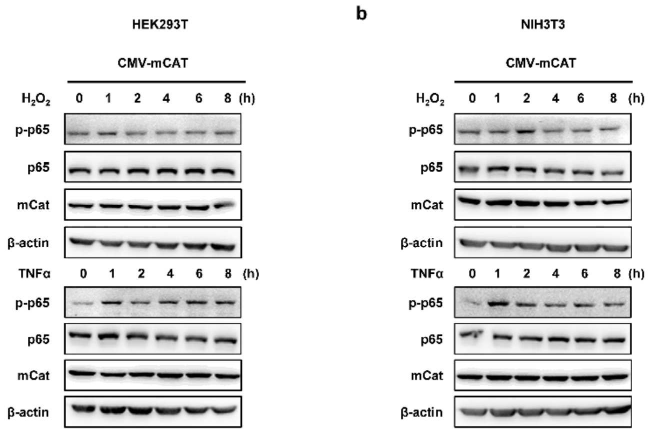

Figure. S4. The mCat expression of CMV-mCAT system responses to stress.

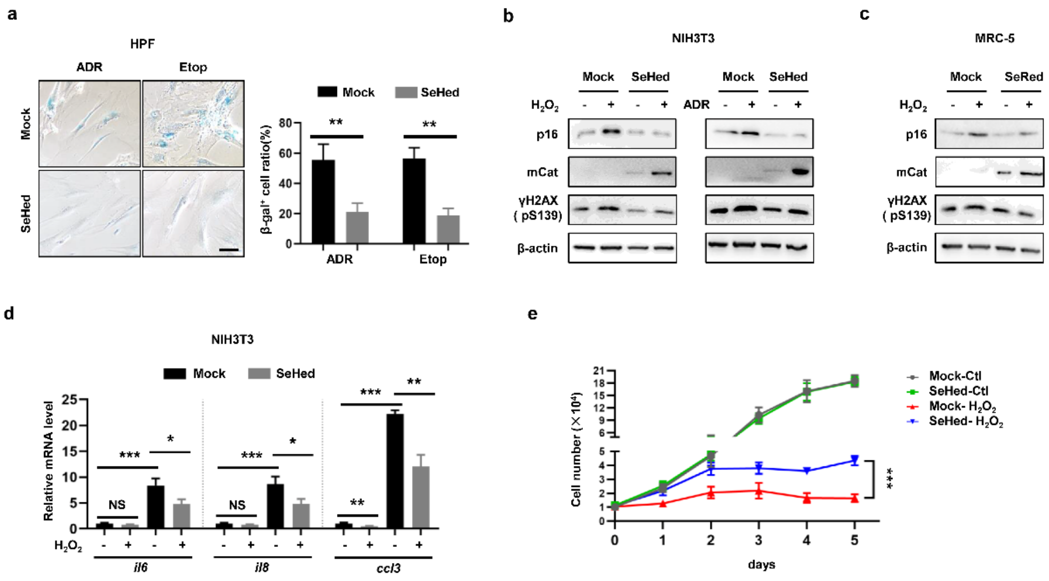

Figure. S5. SeHed alleviates cellular senescence.

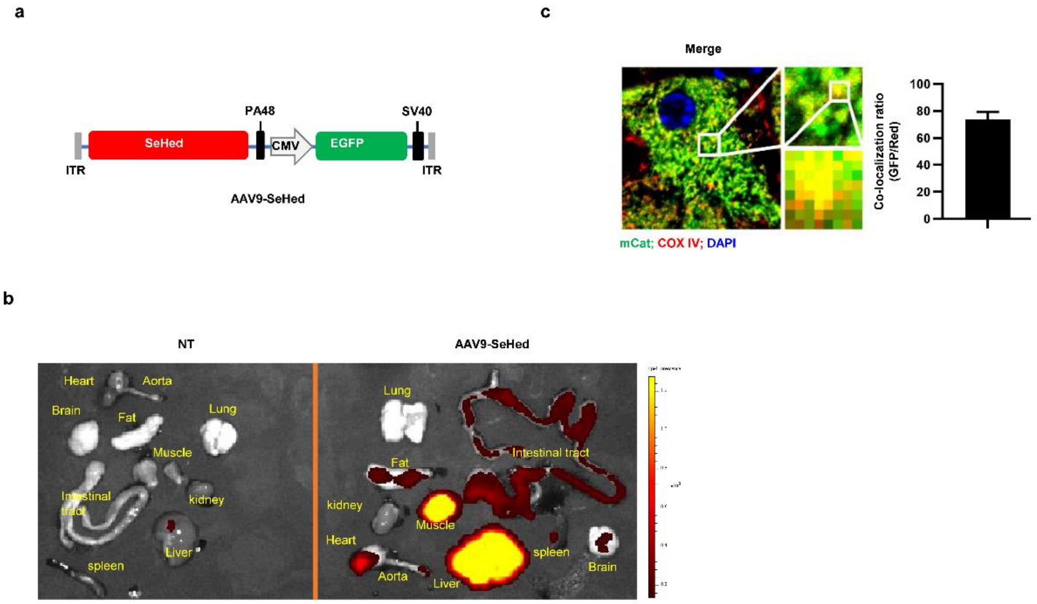

Figure. S6. The distribution and the localization of AAV9-SeHed.

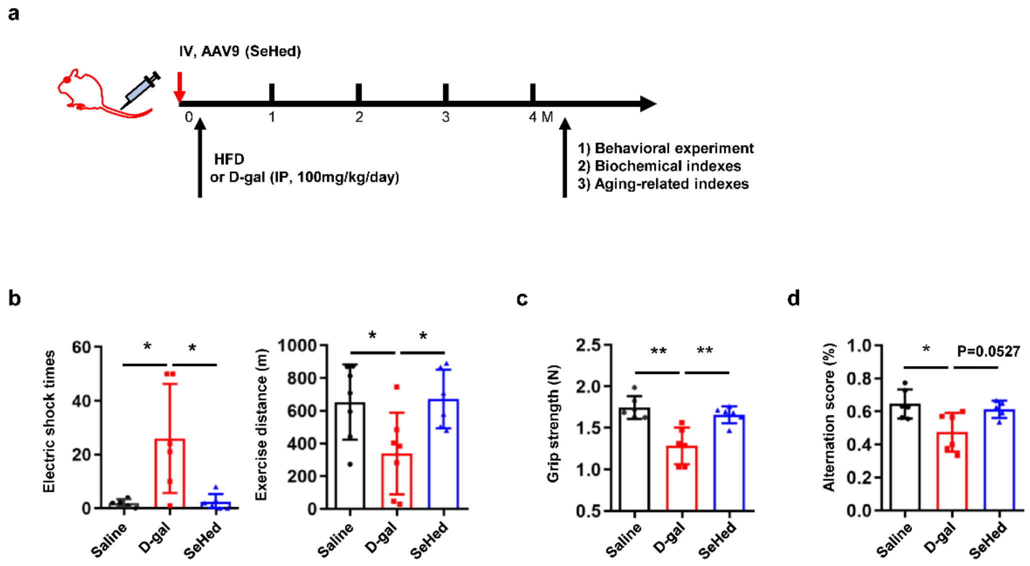

Figure. S7. SeHed improves age-related behavioral phenotypes in progeria mice model.

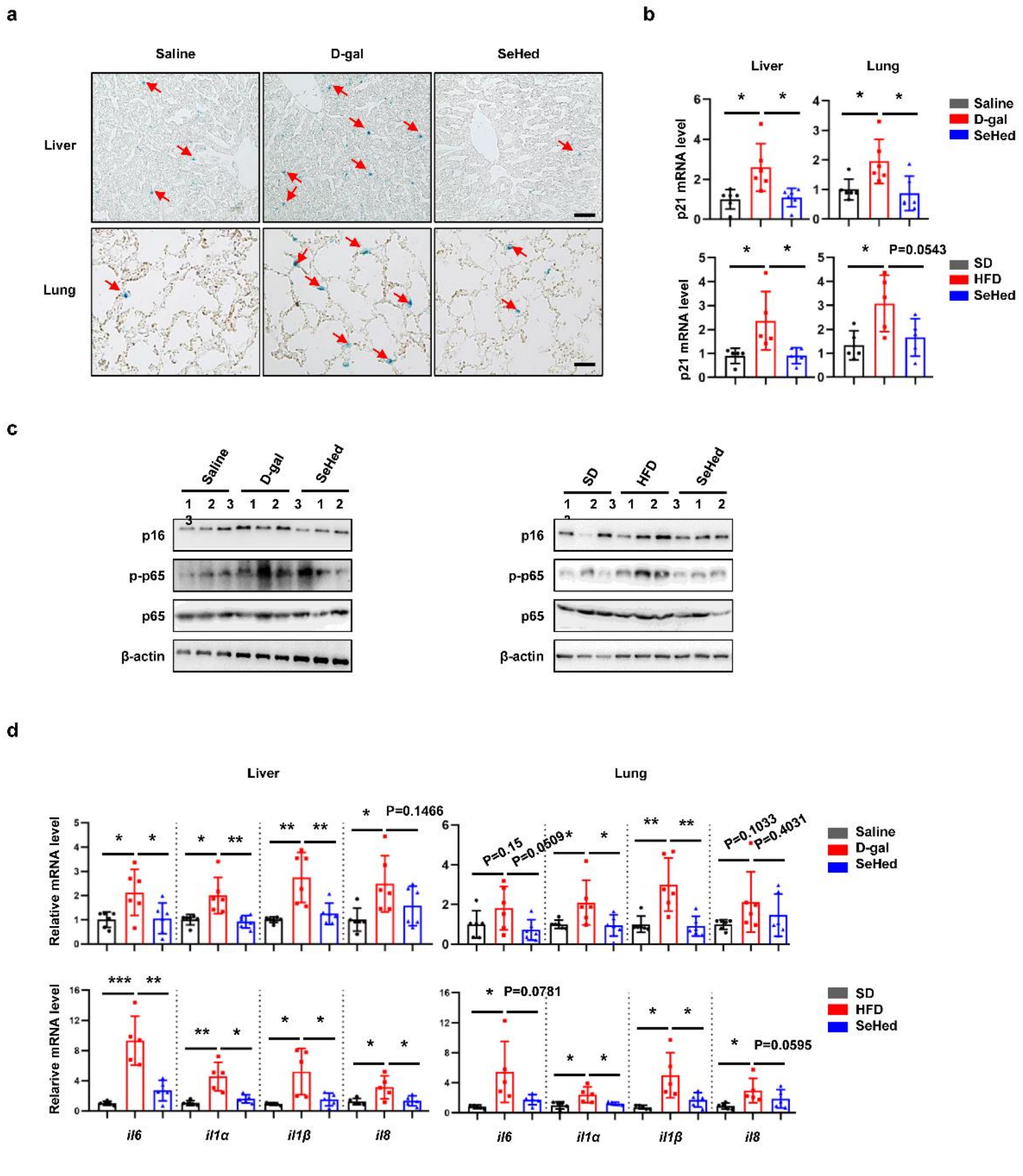

Figure. S8. SeHed alleviates tissues aging in progeria mice model and metabolic disorder model.

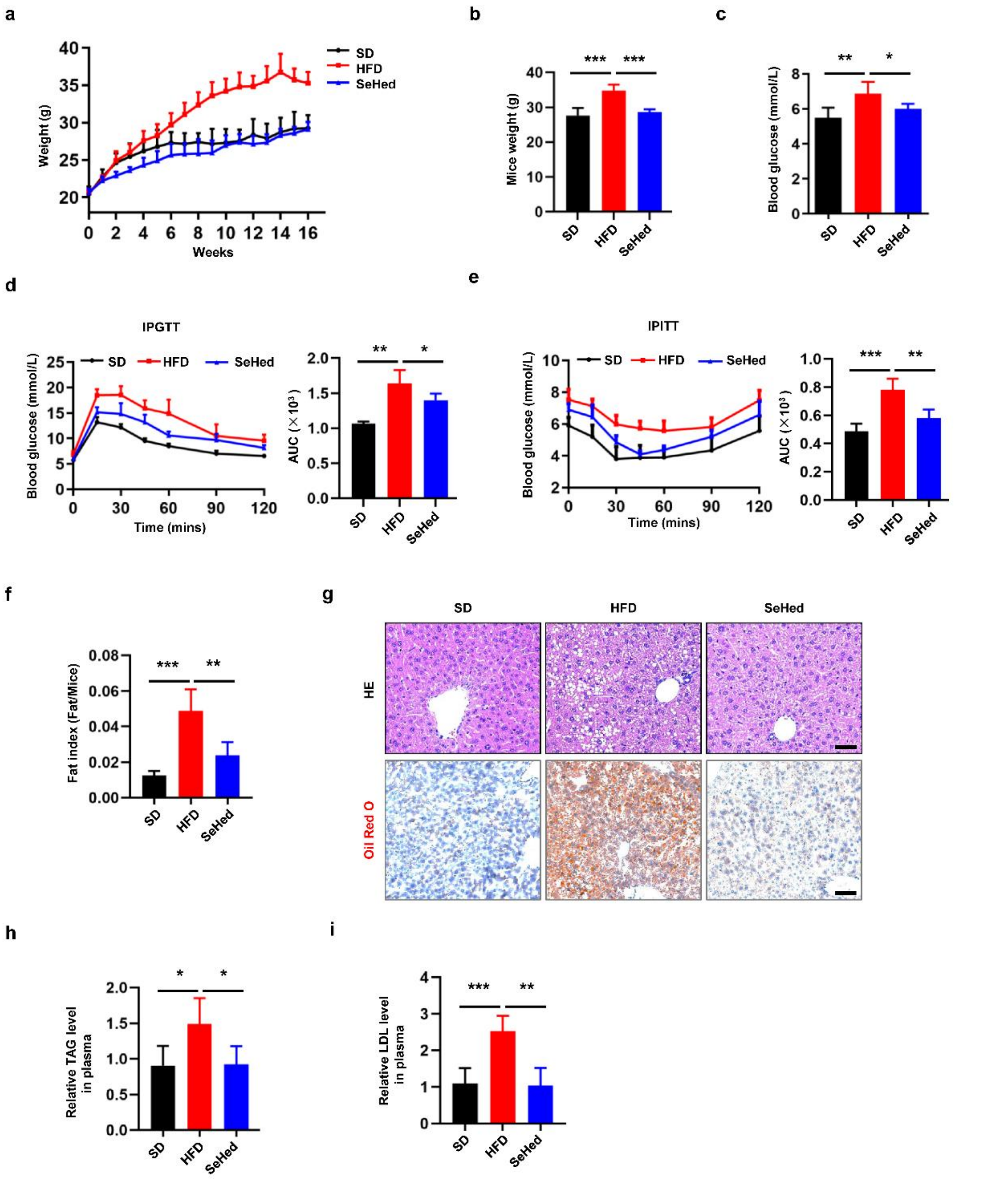

Figure. S9. SeHed prevents HFD-induced obesity and metabolic dysfunction.

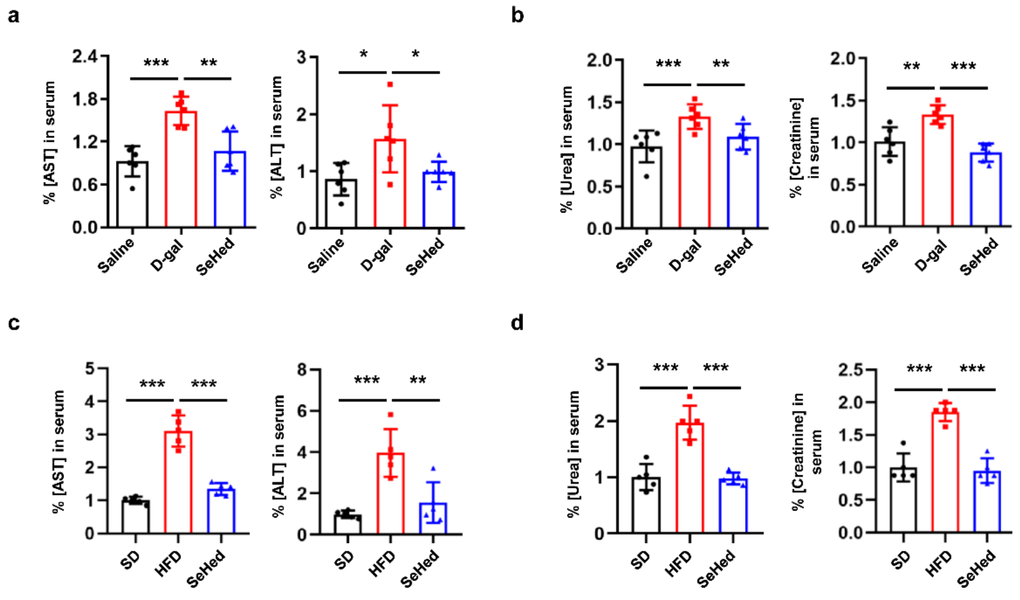

Figure. S10. SeHed alleviates liver and kidney damage in progeria mice model and in metabolic disorder model.

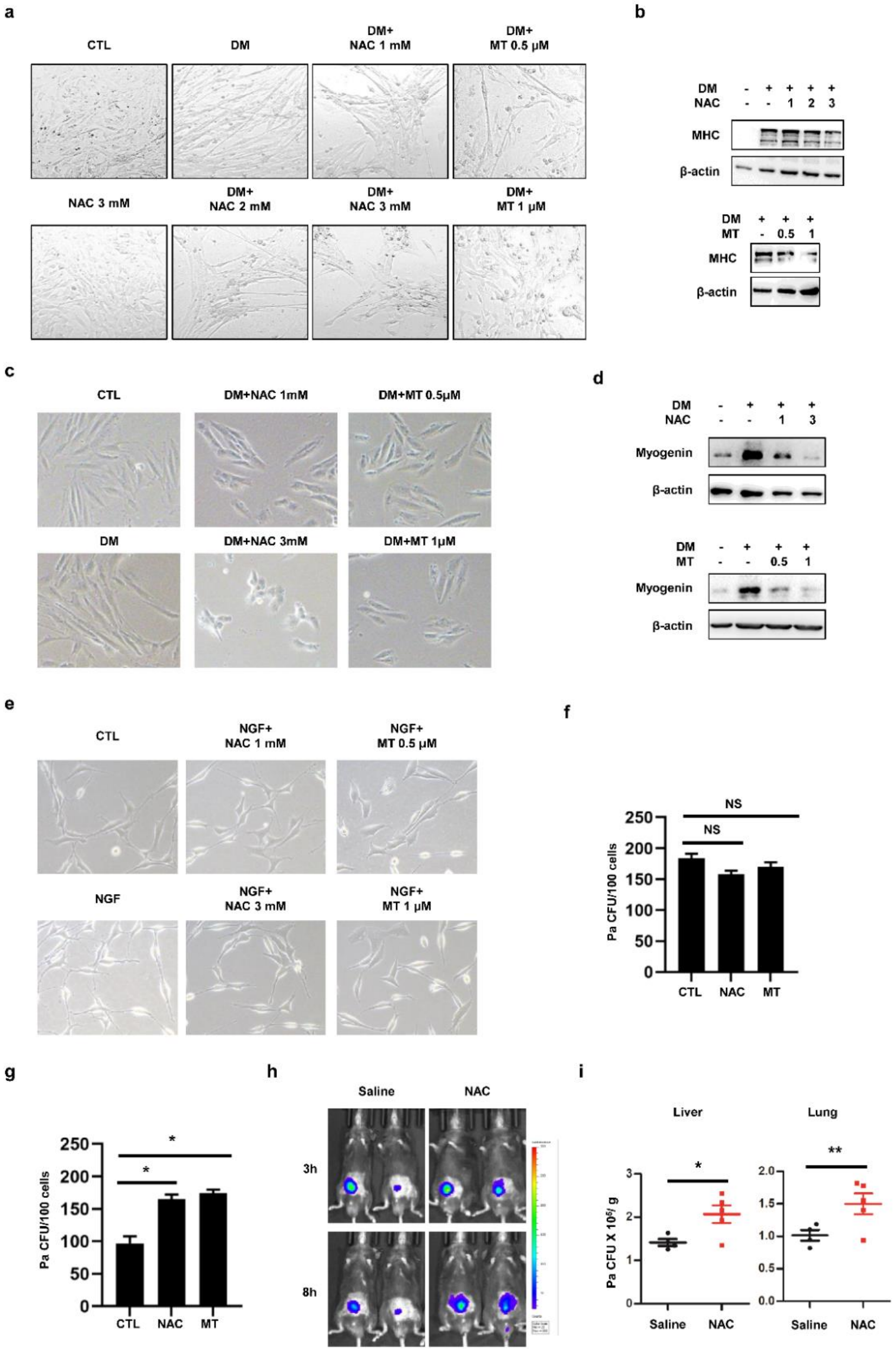

Figure. S11. Antioxidants impairs capacities for stem cell differentiation and bacterial infection resistance.

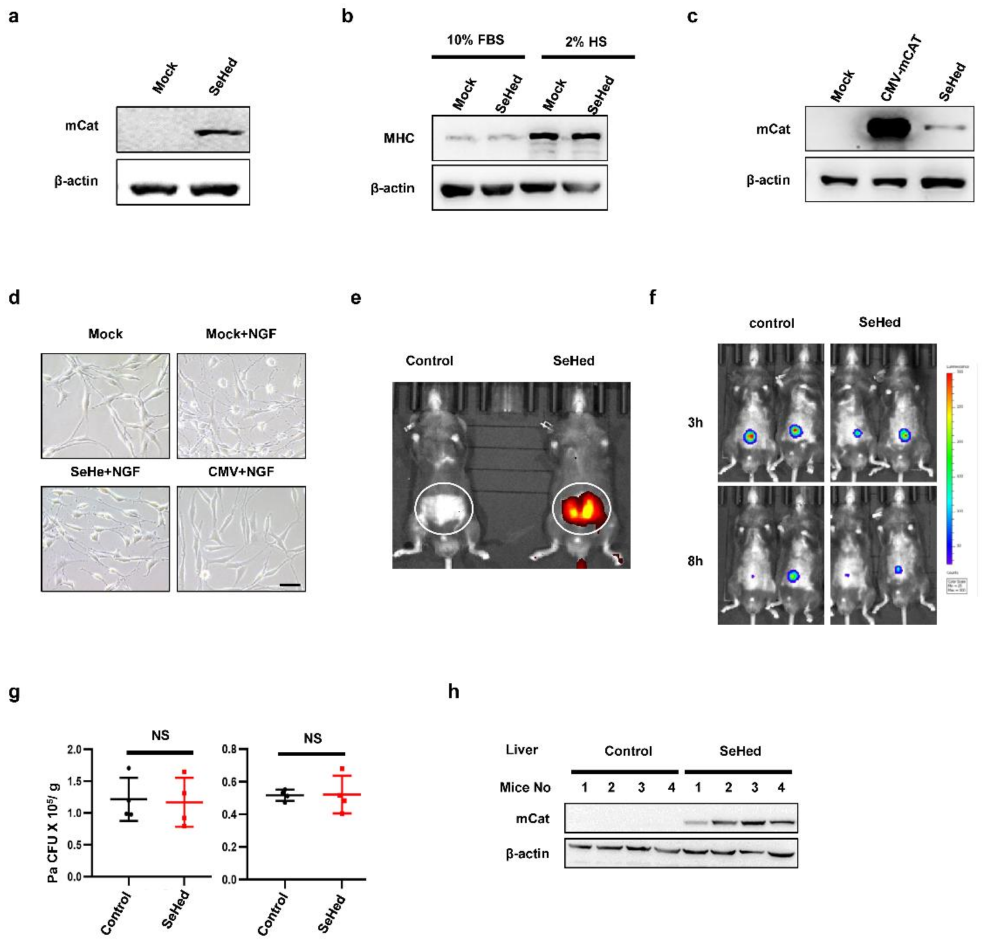

Figure. S12. SeHed does not impairs stem cell differentiation in vitro and bacterial infection resistance in vivo.

Primers	Forward primer	Reverse primer
18s	TTGACGGAAGGGCACCACCAG	GCACCACCACCCACGGAATCG
m*il6*	CTGCAAGAGACTTCCATCCAG	AGTGGTATAGACAGGTCTGTTGG
m*il8*	CTGGTCCATGCTCCTGCTG	GGACGGACGAAGATGCCTAG
m*ccl3*	TTCTCTGTACCATGACACTCTGC	CGTGGAATCTTCCGGCTGTAG
m*il1β*	GCAACTGTTCCTGAACTCAACT	ATCTTTTGGGGTCCGTCAACT
m*il1α*	CGAAGACTACAGTTCTGCCATT	GACGTTTCAGAGGTTCTCAGAG
m*tnfα*	GAAGATGATCTGAGTGTGAGGGT	GCAATACGGACTTGCTCACAGA
mCat	ATGCTGTTTAATCTGAGGATC	TGTCTCCTACTGGGTTACC
m*p21*	GTGGCCTTGTCGCTGTCTT	GCGCTTGGAGTGATAGAAATCTG

Table. S1. The primers for the experiment.
