# Supplementary figures and images for: SeHed, a novel gene expression system with stress-evoked hydrogen peroxide elimination property and anti-aging effect
Source: Signal Transduct Target Ther. 2022 Jul 15;7:235. doi: 10.1038/s41392-022-01047-2 (PMC9283520; doi:10.1038/s41392-022-01047-2)

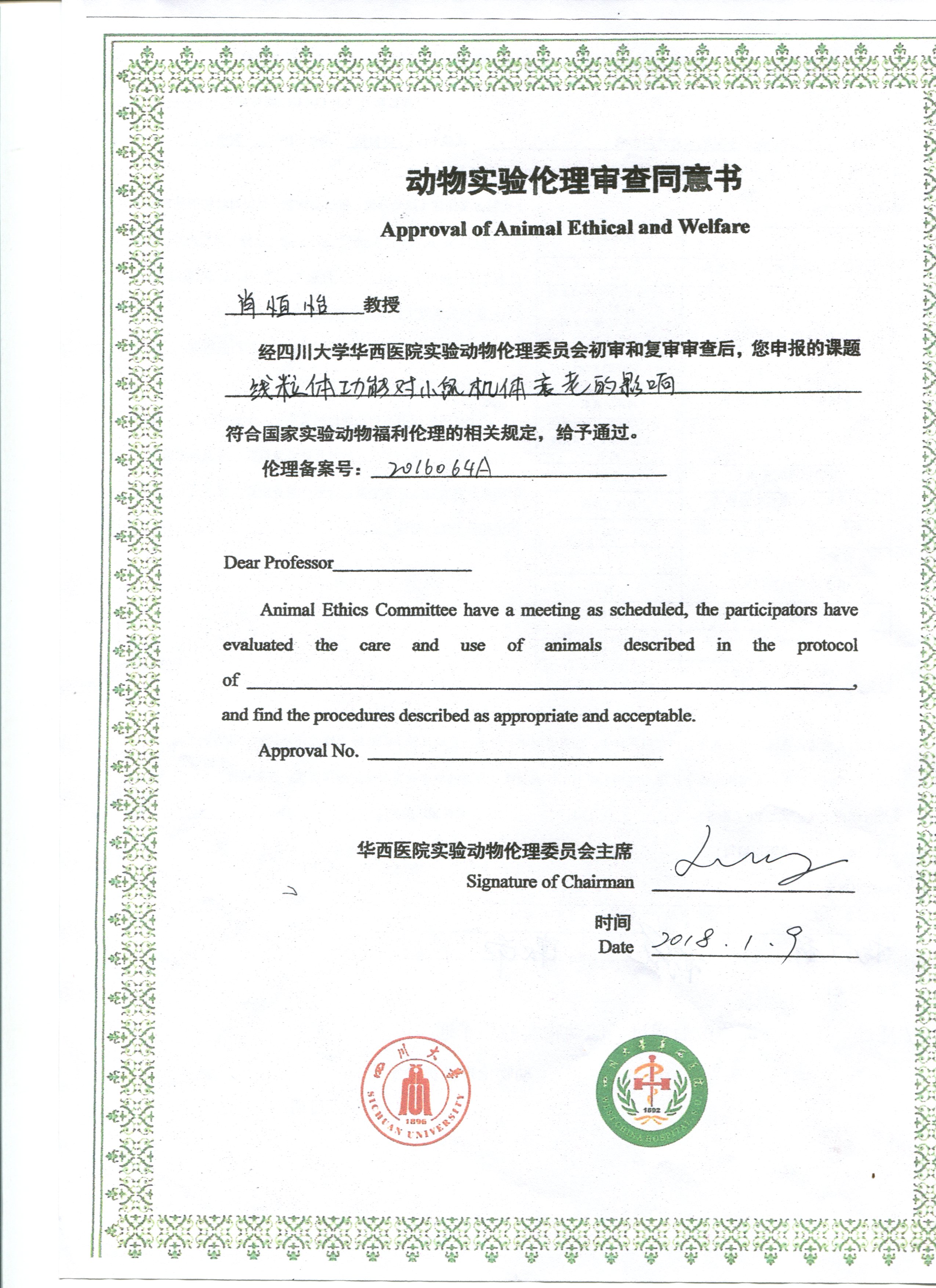

Supplement: Supplementary file 3 — Ethics declarations [file 41392_2022_1047_MOESM3_ESM.jpg]

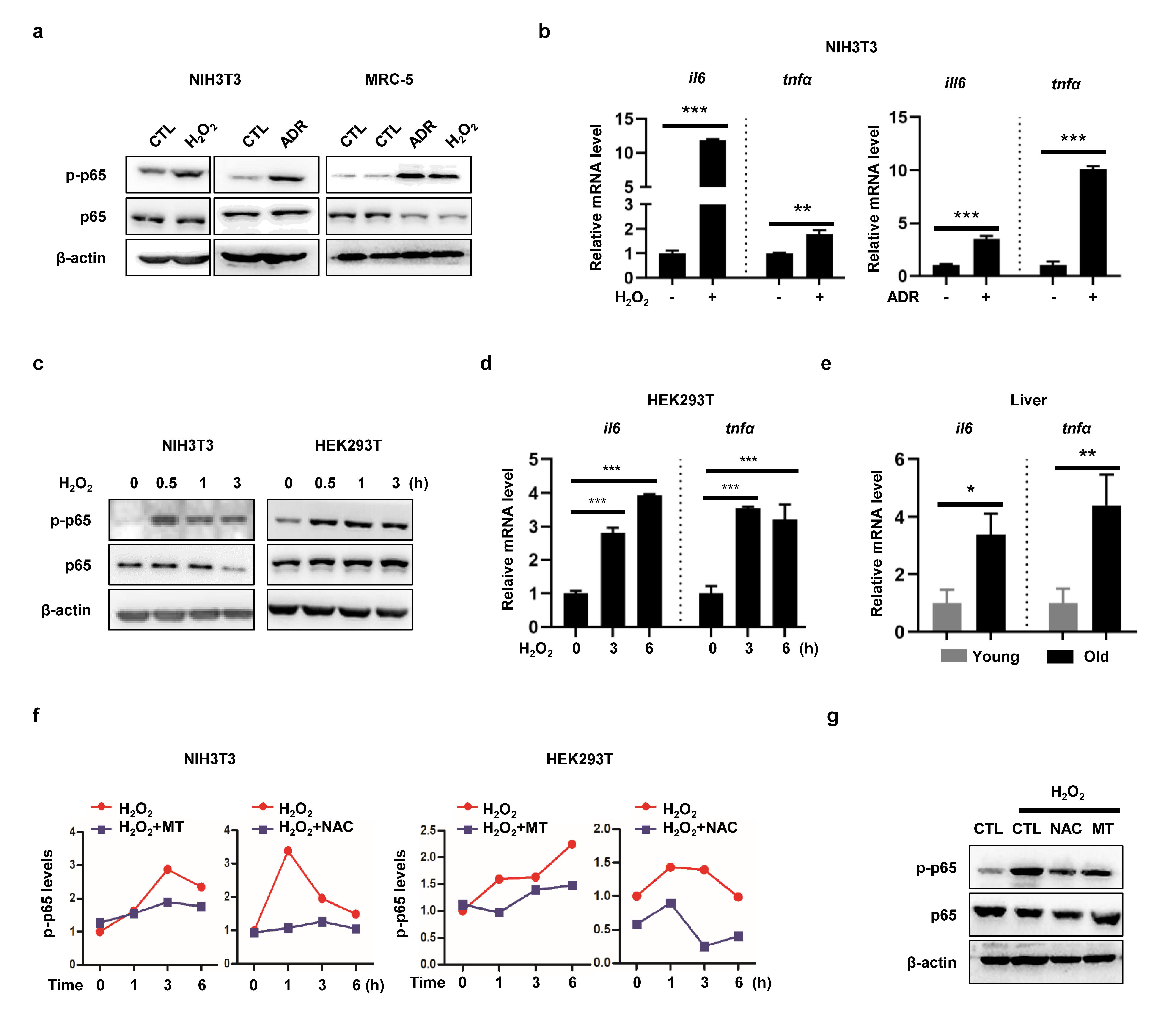

Supplement: Supplementary file 4 — Supplementary Figures 1 [file 41392_2022_1047_MOESM4_ESM.tif]

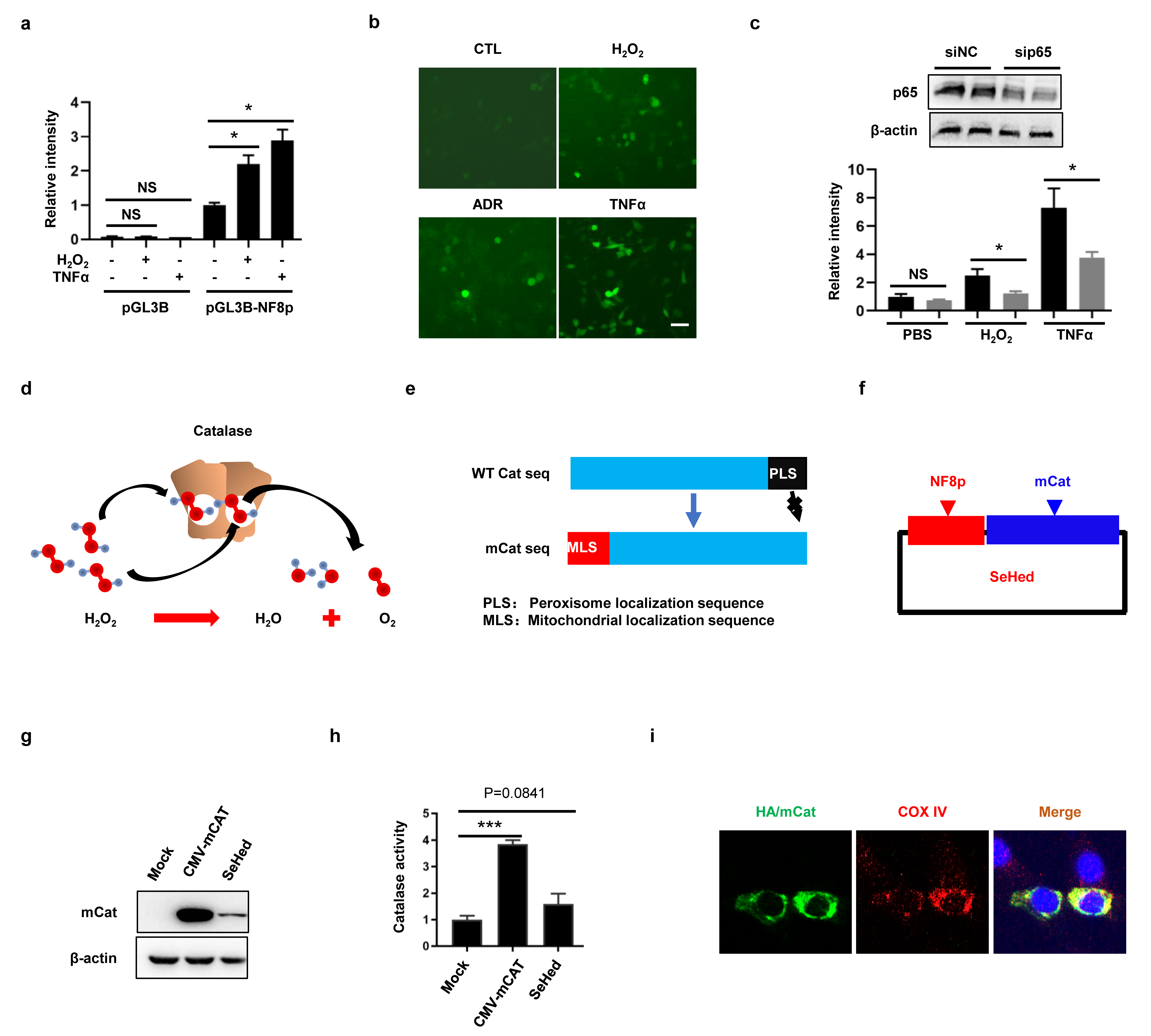

Supplement: Supplementary file 5 — Supplementary Figures 2 [file 41392_2022_1047_MOESM5_ESM.tif]

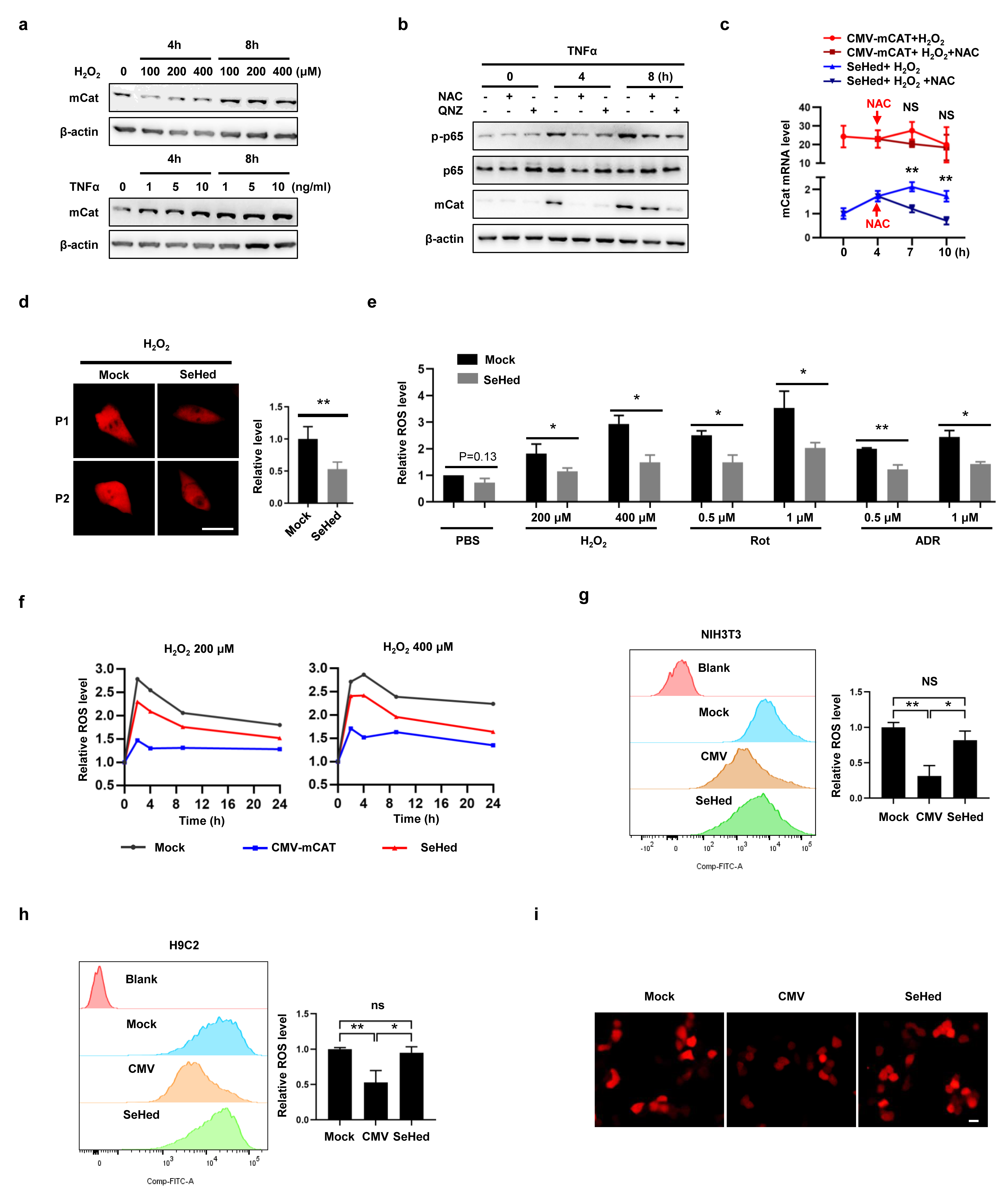

Supplement: Supplementary file 6 — Supplementary Figures 3 [file 41392_2022_1047_MOESM6_ESM.tif]

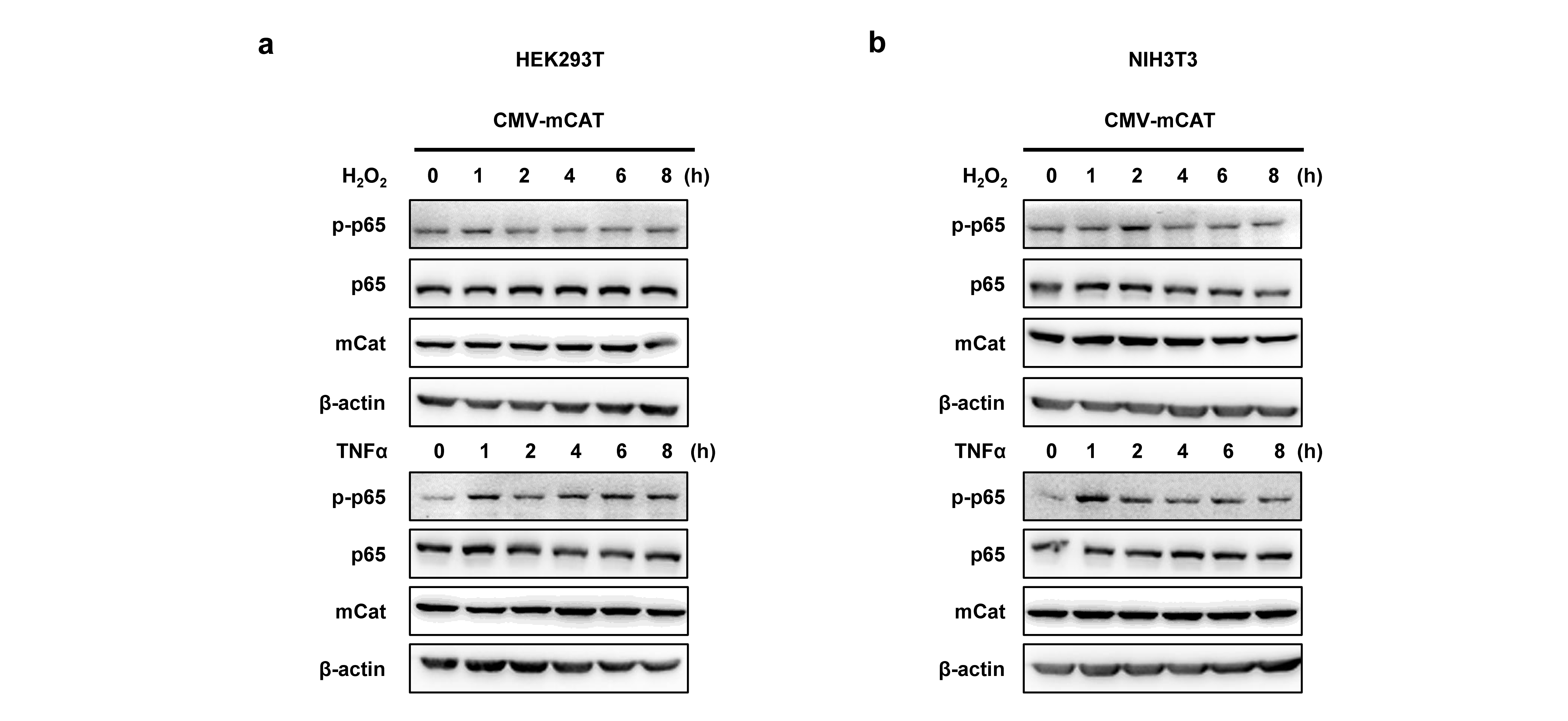

Supplement: Supplementary file 7 — Supplementary Figures 4 [file 41392_2022_1047_MOESM7_ESM.tif]

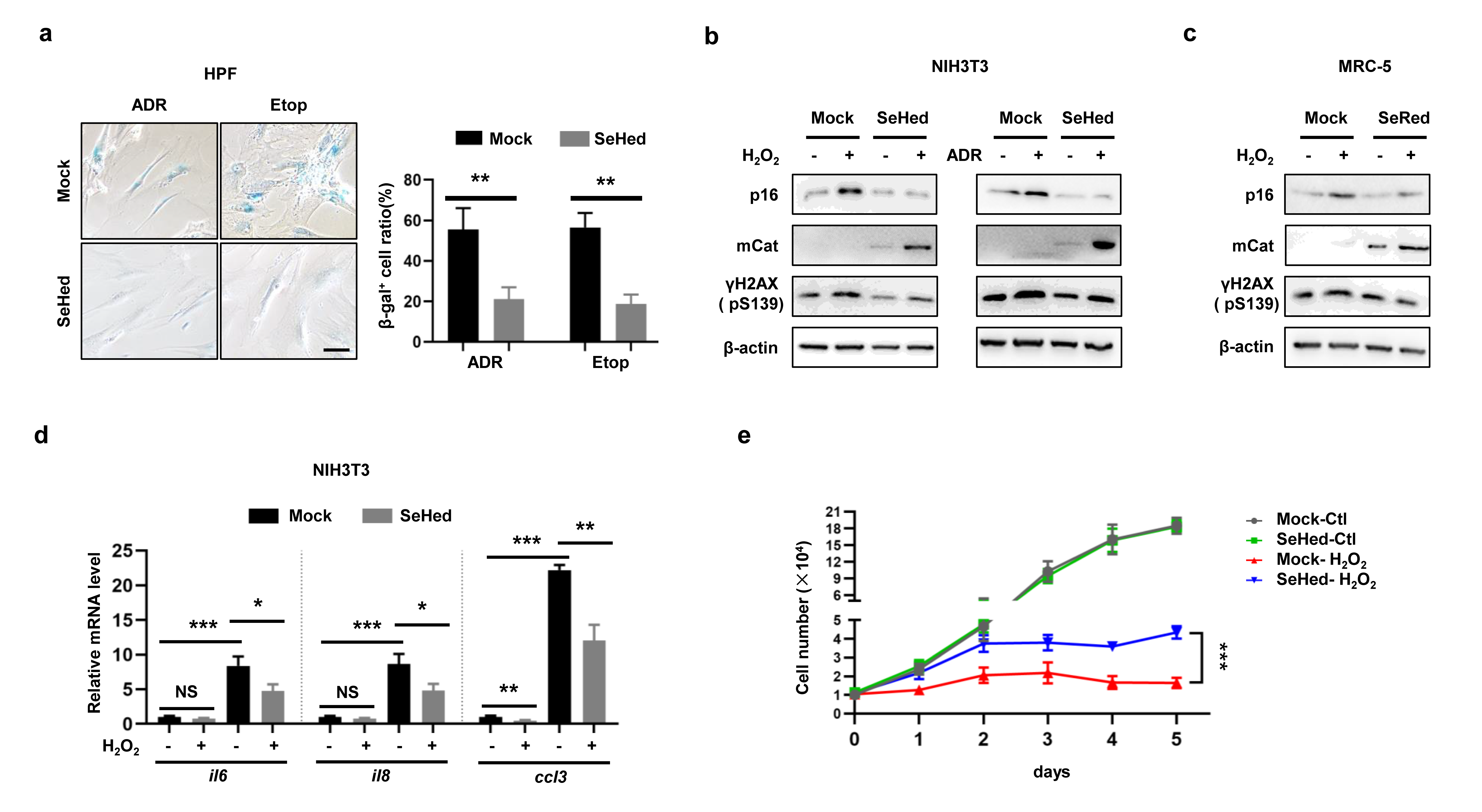

Supplement: Supplementary file 8 — Supplementary Figures 5 [file 41392_2022_1047_MOESM8_ESM.tif]

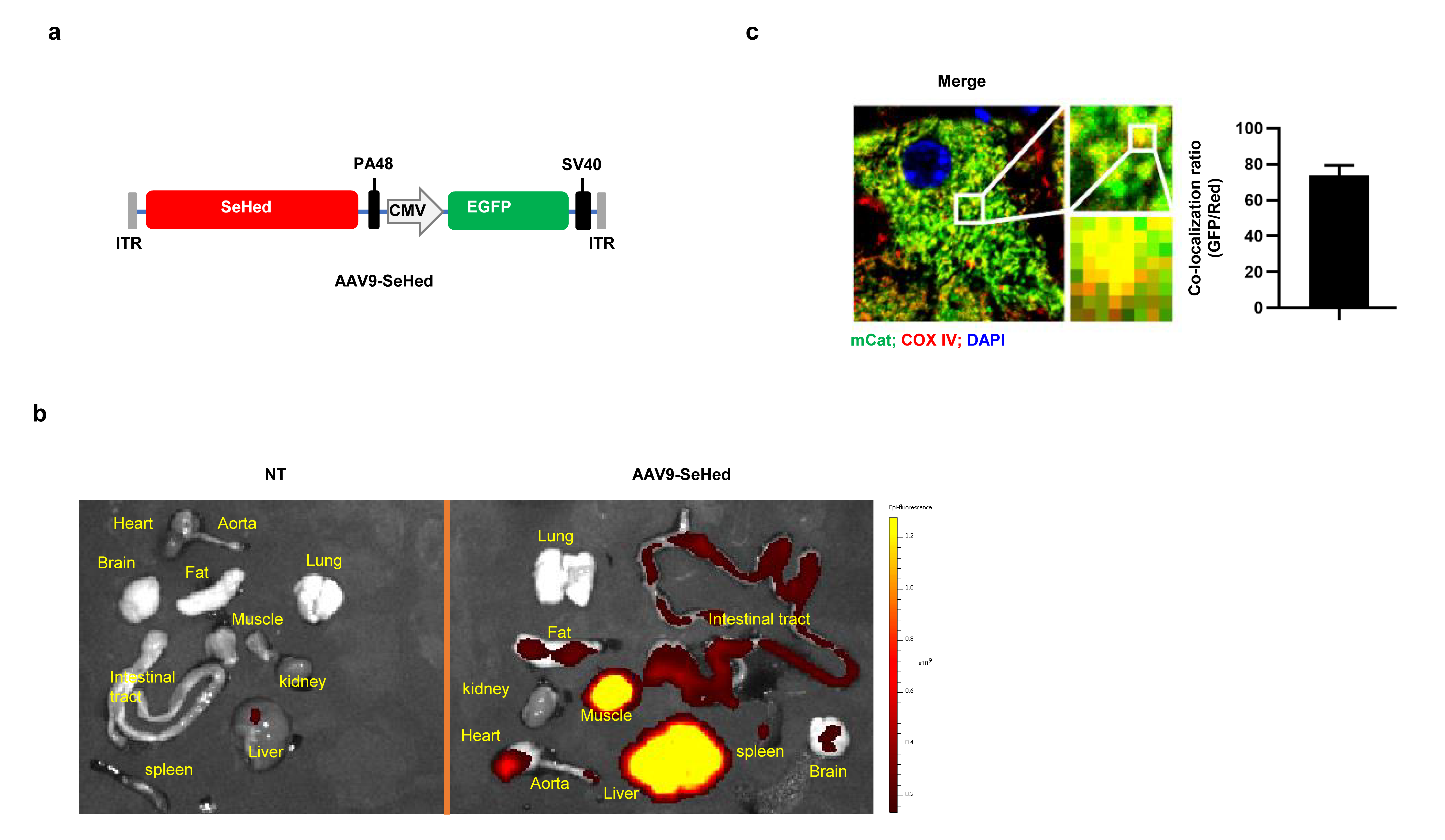

Supplement: Supplementary file 9 — Supplementary Figures 6 [file 41392_2022_1047_MOESM9_ESM.tif]

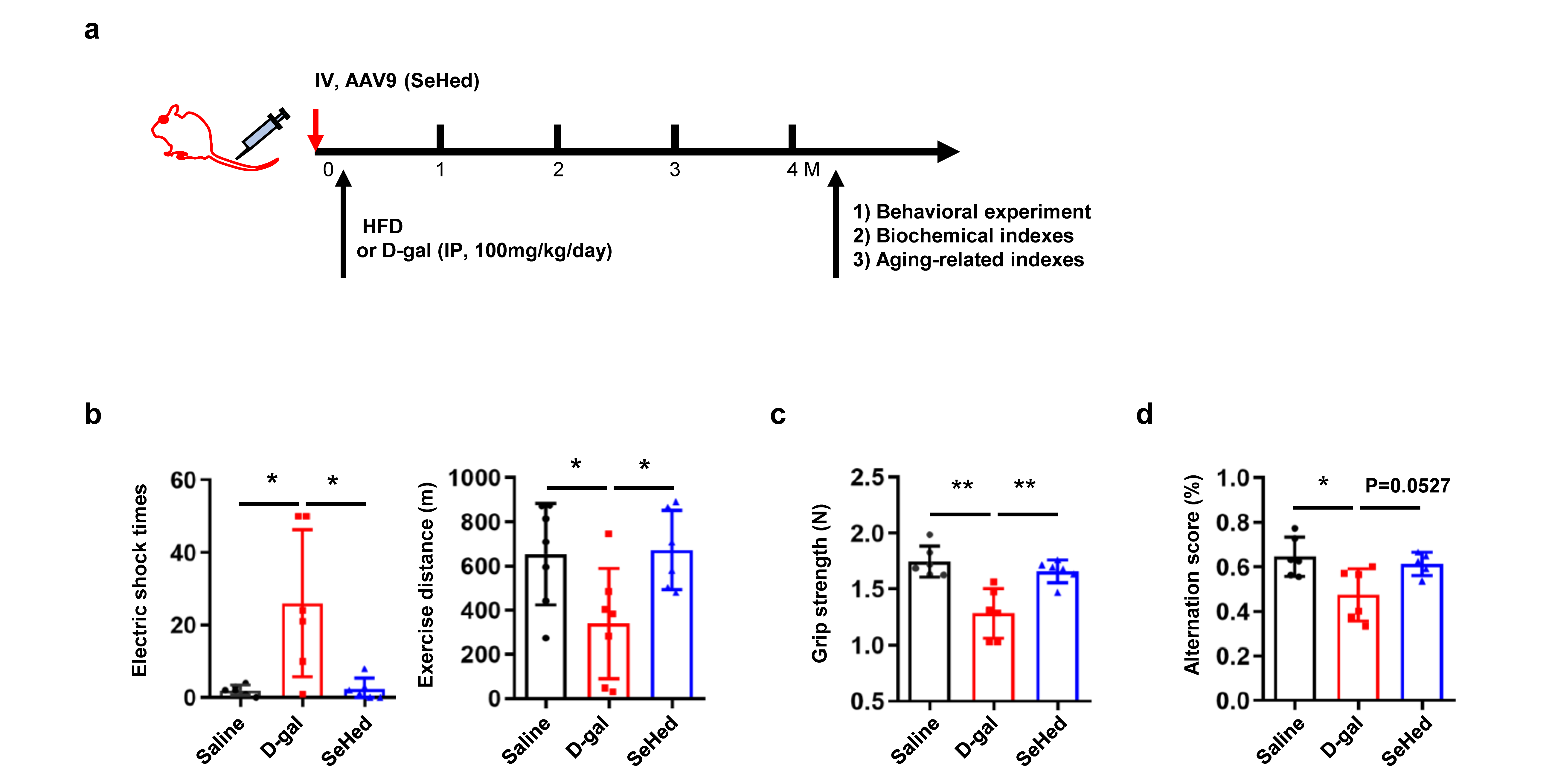

Supplement: Supplementary file 10 — Supplementary Figures 7 [file 41392_2022_1047_MOESM10_ESM.tif]

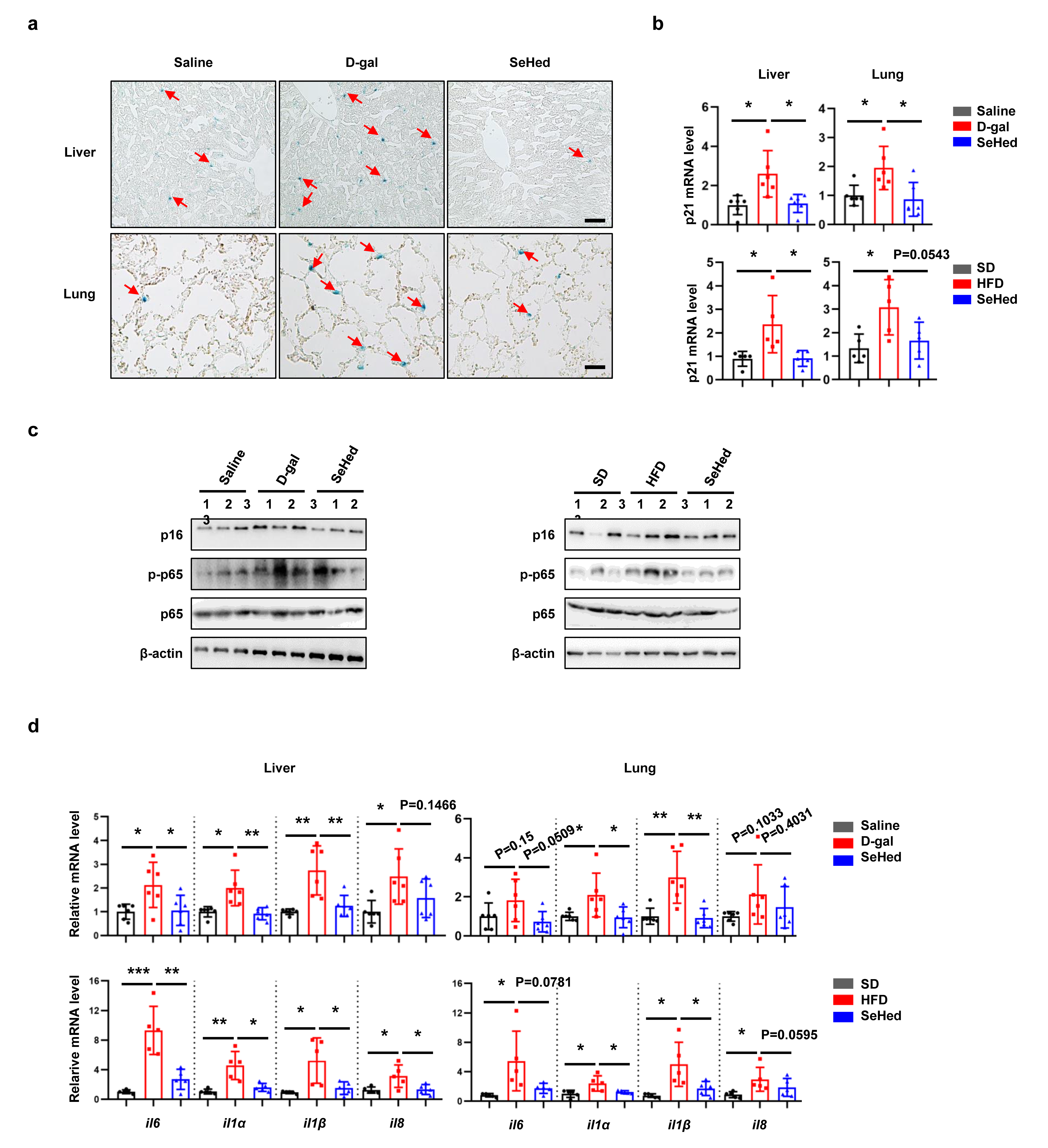

Supplement: Supplementary file 11 — Supplementary Figures 8 [file 41392_2022_1047_MOESM11_ESM.tif]

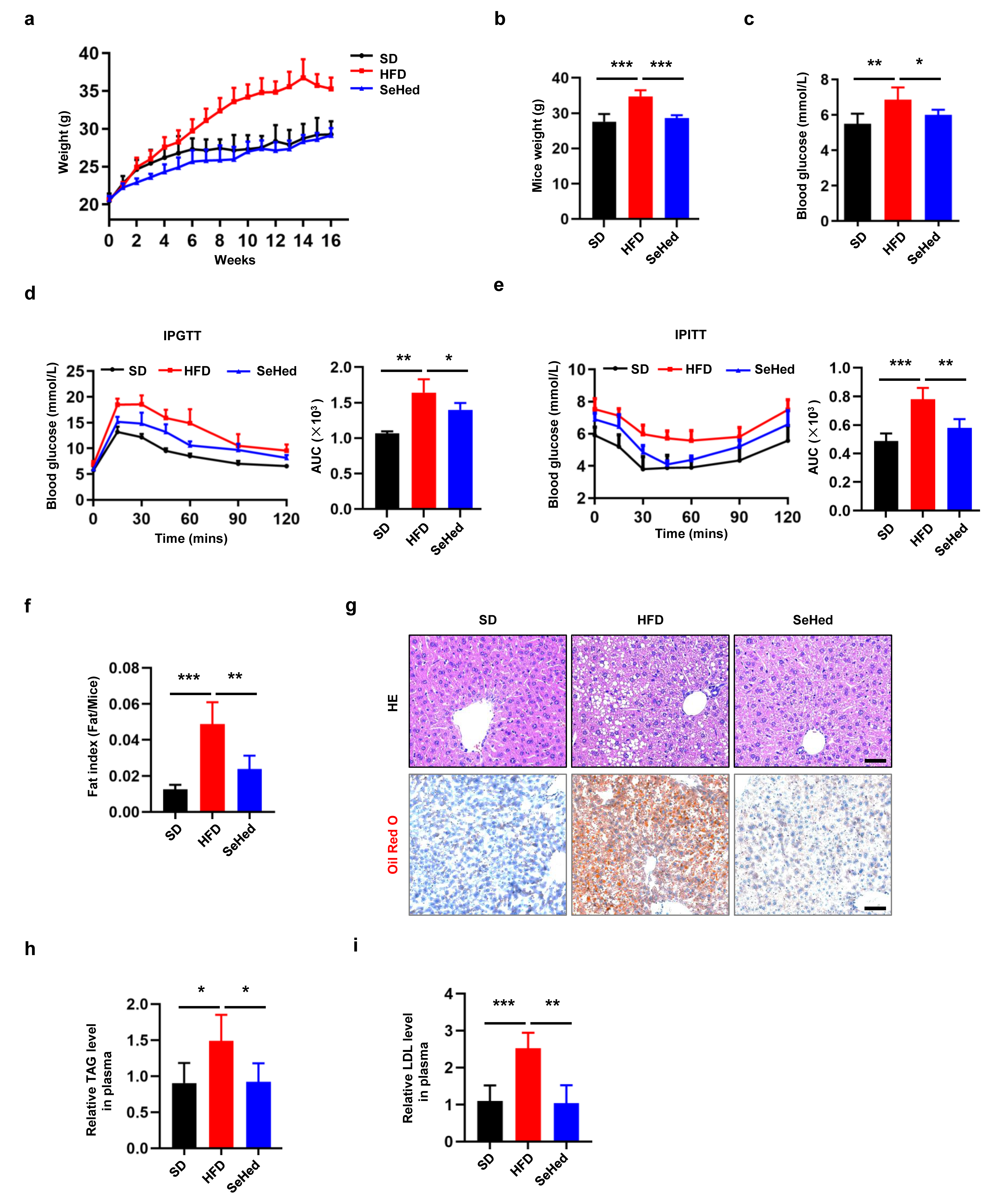

Supplement: Supplementary file 12 — Supplementary Figures 9 [file 41392_2022_1047_MOESM12_ESM.tif]

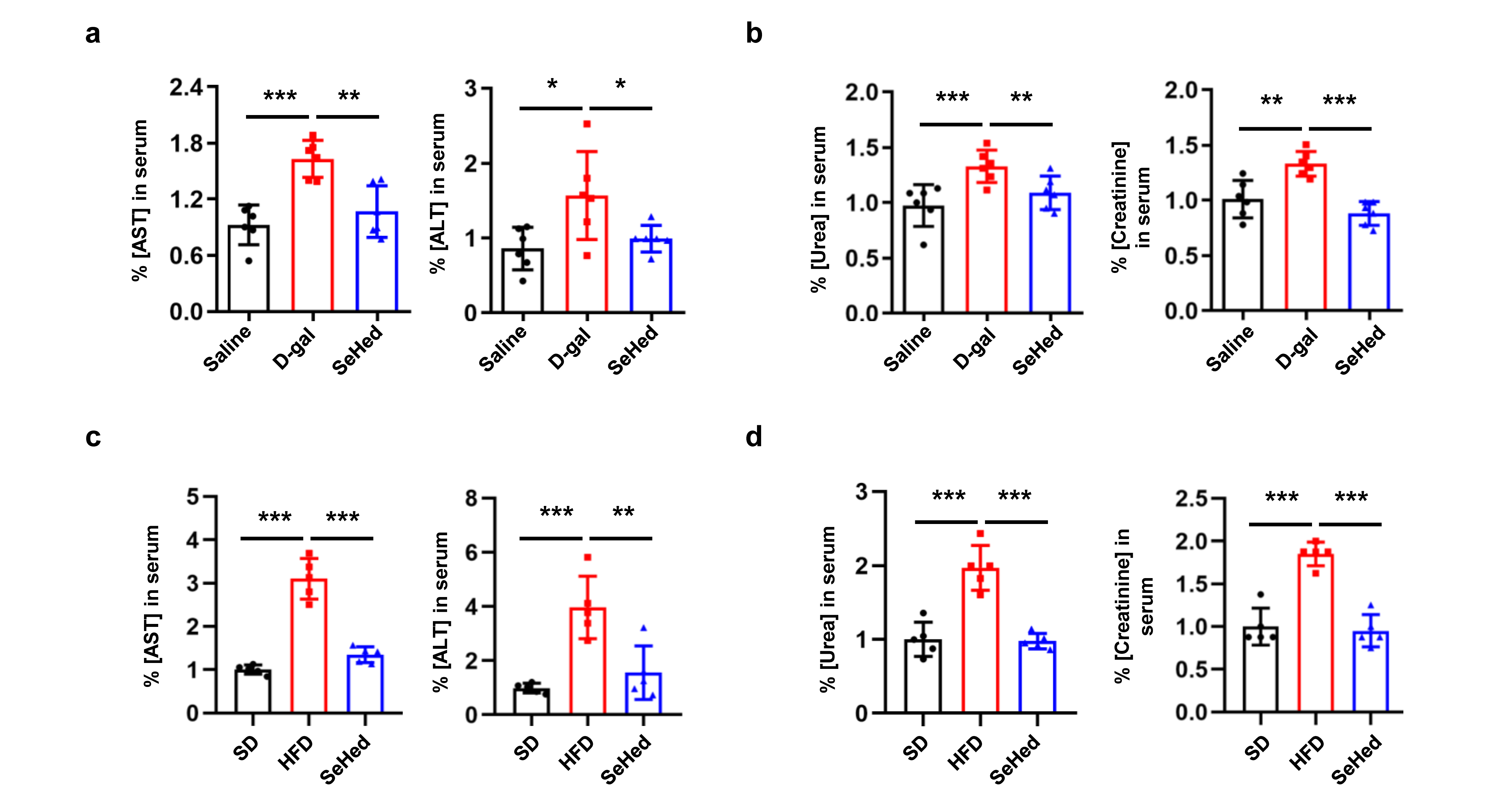

Supplement: Supplementary file 13 — Supplementary Figures 10 [file 41392_2022_1047_MOESM13_ESM.tif]

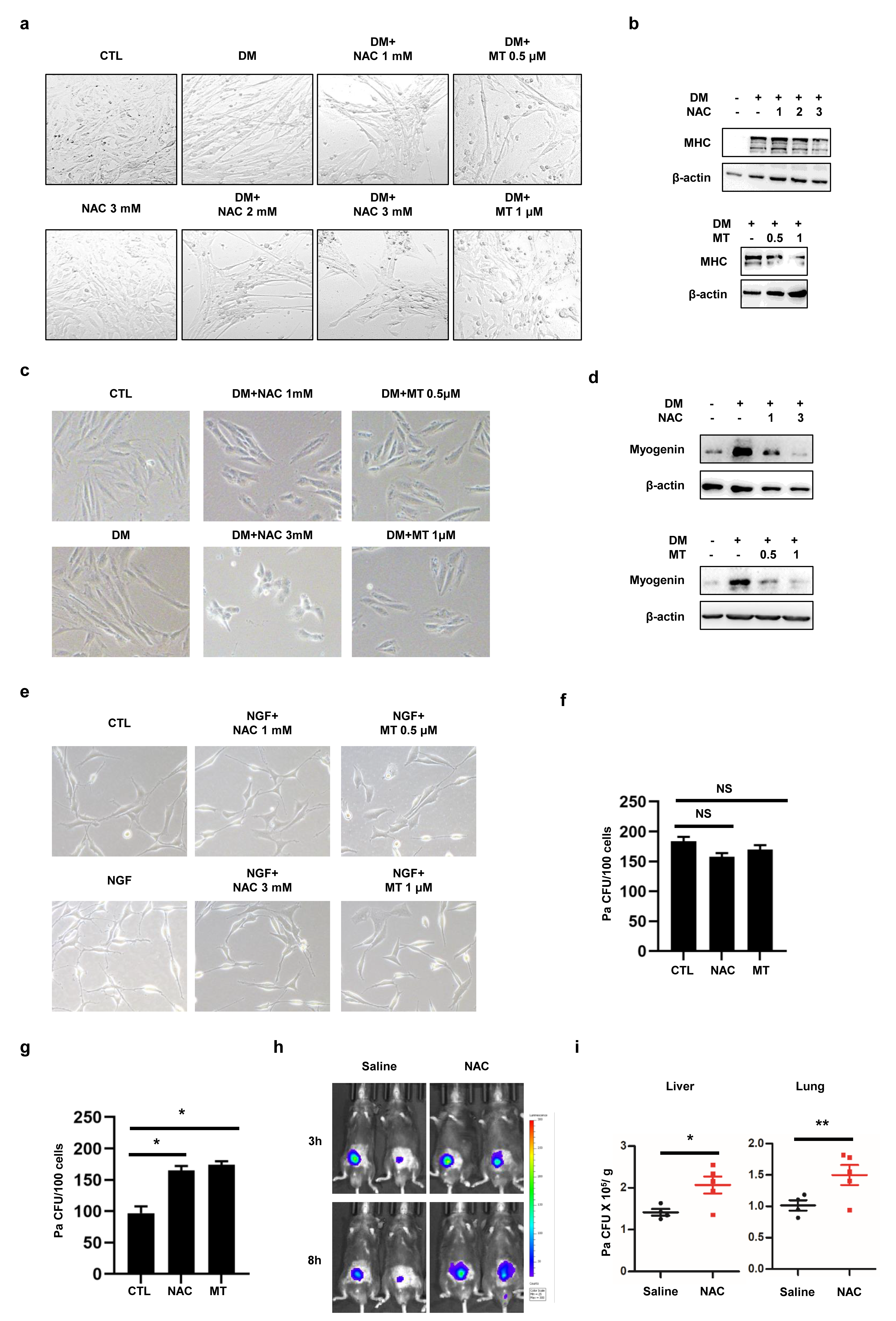

Supplement: Supplementary file 14 — Supplementary Figures 11 [file 41392_2022_1047_MOESM14_ESM.tif]

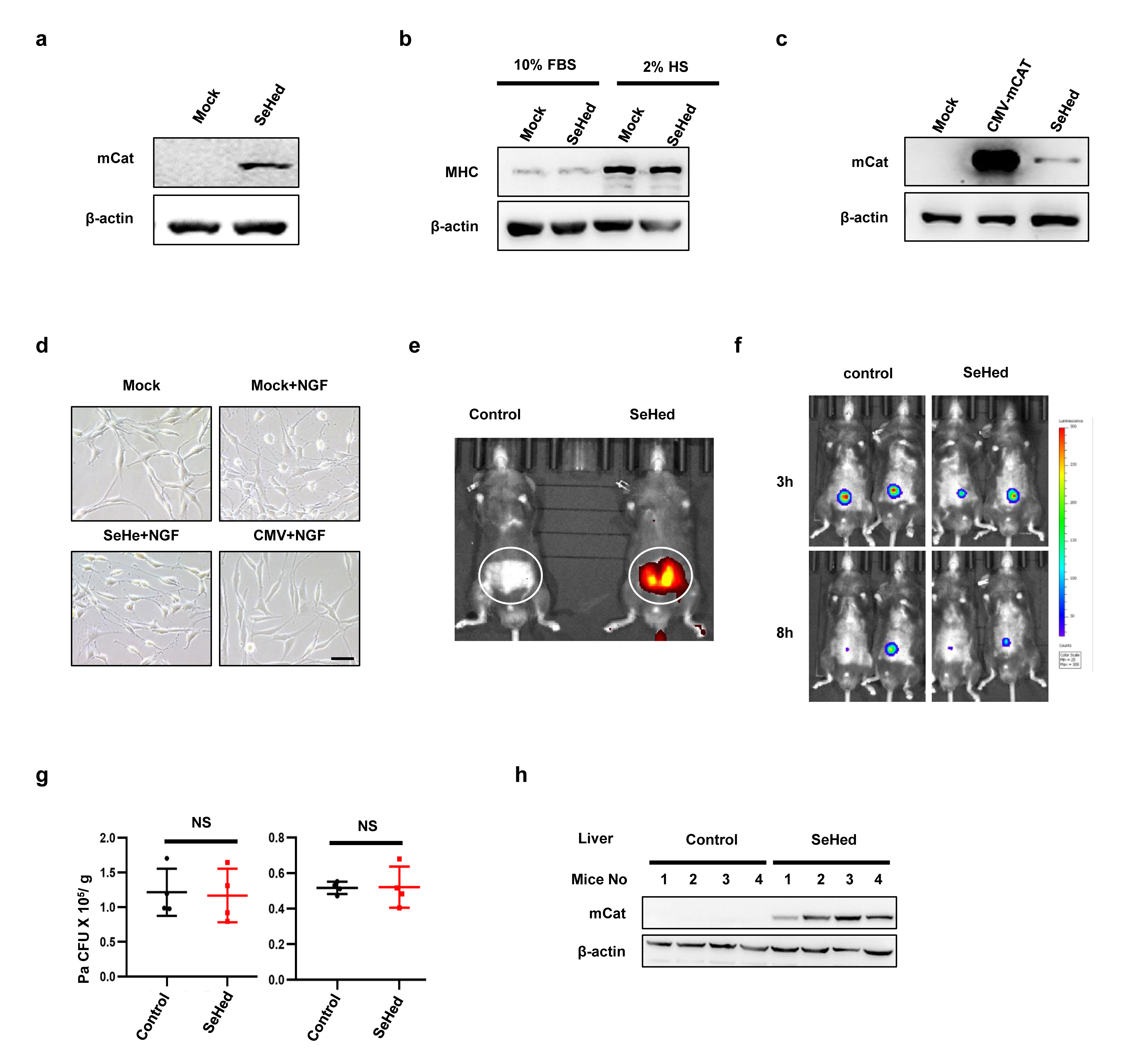

Supplement: Supplementary file 15 — Supplementary Figures 12 [file 41392_2022_1047_MOESM15_ESM.tif]
